# Automated hierarchical classification of protein domain subfamilies based on functionally-divergent residue signatures

**DOI:** 10.1186/1471-2105-13-144

**Published:** 2012-06-22

**Authors:** Andrew F Neuwald, Christopher J Lanczycki, Aron Marchler-Bauer

**Affiliations:** 1 Institute for Genome Sciences and Department of Biochemistry & Molecular Biology, University of Maryland School of Medicine, BioPark II, Room 617, 801 West Baltimore St, Baltimore, MD 21201, USA; 2 National Center for Biotechnology Information; National Library of Medicine, National Institutes of Health, Bethesda, MD 20894, USA

## Abstract

**Background:**

The NCBI Conserved Domain Database (CDD) consists of a collection of multiple sequence alignments of protein domains that are at various stages of being manually curated into evolutionary hierarchies based on conserved and divergent sequence and structural features. These domain models are annotated to provide insights into the relationships between sequence, structure and function via web-based BLAST searches.

**Results:**

Here we automate the generation of conserved domain (CD) hierarchies using a combination of heuristic and Markov chain Monte Carlo (MCMC) sampling procedures and starting from a (typically very large) multiple sequence alignment. This procedure relies on statistical criteria to define each hierarchy based on the conserved and divergent sequence patterns associated with protein functional-specialization. At the same time this facilitates the sequence and structural annotation of residues that are functionally important. These statistical criteria also provide a means to objectively assess the quality of CD hierarchies, a non-trivial task considering that the protein subgroups are often very distantly related—a situation in which standard phylogenetic methods can be unreliable. Our aim here is to automatically generate (typically sub-optimal) hierarchies that, based on statistical criteria and visual comparisons, are comparable to manually curated hierarchies; this serves as the first step toward the ultimate goal of obtaining optimal hierarchical classifications. A plot of runtimes for the most time-intensive (non-parallelizable) part of the algorithm indicates a nearly linear time complexity so that, even for the extremely large Rossmann fold protein class, results were obtained in about a day.

**Conclusions:**

This approach automates the rapid creation of protein domain hierarchies and thus will eliminate one of the most time consuming aspects of conserved domain database curation. At the same time, it also facilitates protein domain annotation by identifying those pattern residues that most distinguish each protein domain subgroup from other related subgroups.

## Background

In order to provide rapid and sensitive annotation for protein sequences, including direct links to structural and functional information, the National Center for Biotechnology Information (NCBI) initiated the Conserved Domain Database (CDD) [1] —a collection of position-specific scoring matrices (PSSMs) (essentially HMM profiles [2]) that are derived from protein multiple sequence alignments. As a result, web-based BLAST searches now include a search of the CDD, which allows users to visualize multiple sequence alignments and (via the NCBI Cn3D viewer [3]) structures of proteins sharing significant homology to the query and, within those alignments, key catalytic and ligand-binding residues. Thus BLAST searches linked to the CDD provide additional clues to the function and underlying mechanism of the query protein and are thereby often more informative, faster and more sensitive than searching against millions of individual protein sequences.

The CDD is comprised of domain models either manually curated at the NCBI or imported from other alignment collections such as PFam [4], SMART [5], and TIGRFAM [6]. A central and unique feature of the CDD is that related domains are organized into hierarchies when evidence exists to support that tree as a representation of the molecular evolution of the protein class. A significant bottleneck in the CDD pipeline is the curation of these hierarchies and the manual annotation of the corresponding profiles for functionally important residues (as gleaned from the biochemical literature). In order to begin automating this process, here we describe statistically-rigorous procedures for automated creation of conserved domain (CD) hierarchies and for annotation of the corresponding profile alignments. These procedures do automatically, based on objective, empirical criteria, what the CDD resource group and similar groups currently do manually, based on classifications that have been established in the published literature, on phylogenetic and structural analysis and, to some degree, on subjective judgments. Our focus here is to obtain heuristically an initial (presumably sub-optimal) CD hierarchy starting from a typically very large multiple sequence alignment for an entire protein class whose domain boundaries remain fixed. To do this we utilize procedures that obtain both subgroup assignments for aligned sequences and corresponding discriminating patterns associated with protein functional-divergence.

The automated annotation of functionally critical residues is an important outcome of these proposed procedures: Just as a large enzyme class conserves residues directly involved in catalysis, protein subgroups conserve residues likely involved in subgroup-specific biochemical properties and mechanisms. Our procedures use statistical criteria to glean this biochemical information from patterns of divergent residues among related sequences in a manner similar to the use, by classical geneticists, of statistical criteria to glean information from patterns of divergent traits among related individuals. (To ensure that pattern residues are functionally important, we focus on residues that are conserved across distinct phyla and thus for more than a billion years of evolutionary time). By mapping various categories of pattern residues to corresponding PSSMs, BLAST searches against these improved CD profiles can reveal those residues most likely responsible for the specific biochemical and biophysical properties of a query protein. This can accelerate the pace of biological discovery by enabling researchers to obtain valuable clues regarding as-yet-unidentified protein properties through routine web-based BLAST searches.

Other methods that may be similarly described as addressing the protein subfamily classification problem find sequence clusters either based on pairwise similarity [7-11] or by cutting phylogenetic trees [12-16]. (Phylogenetic trees are, of course, likewise constructed based on sequence and profile similarity scores). Here we take a different approach, namely the hierarchical classification of a protein (domain) class based on functionally-divergent residue signatures. Unlike our approach, many existing methods, though not all (e.g., [15]), generally focus on the narrower problem of identifying orthologs or on the broader problem of clustering a database into unrelated protein classes rather than on constructing a hierarchy of domain profiles for a specific protein class. An approach, which is, in certain respects, similar to the one described here (along with some substantial differences), is the statistical coupling analysis method of Lockless and Ranganathan [17] for detecting sets of correlated residues in protein sequences [18].

Because our approach identifies residues associated with protein functional divergence, it is also related to "functional subtype" prediction (FSP) methods [19-33], but is distinct inasmuch as these related methods typically predict specific residue functions (such as catalytic activity or substrate specificity) that are sufficiently well-understood to allow benchmarking [34,35]. Instead, our approach lets the data itself reveal its most statistically striking properties without making assumptions about the types of residues to be identified. It is further distinguished from each of these related methods in at least several of the following respects: (i) It does not require that the input alignment be partitioned into divergent subsets beforehand; this is unlike many [20-27], though not all [28-31] FSP methods. (ii) It has a rigorous statistical basis. (Though at least two other methods are Bayesian based [36,37]). (iii) It is designed for very large input alignments. (iv) For optimization it relies on Bayesian sampling, which has a solid scientific basis [38]. (We are aware of only one other method [37] with a MCMC sampling component). (v) It separates out unrelated and aberrant sequences automatically. (vi) It can identify multiple categories of co-conserved residues within a given protein. (vii) It addresses concurrently the problems of protein subfamily classification and of identifying residues associated with protein functional divergence. And (viii) it can accomplish all of this automatically starting from a single multiple sequence alignment.

### Problem definition and solution strategy

Here we address the following biological and algorithmic problem: We are given as input a (typically very large) multiple sequence alignment corresponding to a particular protein class. Our objective is to partition this alignment into a tree of sub-alignments, termed a CD hierarchy, each subtree of which corresponds to a sub-alignment of sequences sharing a certain pattern that most distinguishes them from those sequences associated with the parent node of the subtree and with any other subtrees attached to that parent node. We interpret these distinguishing residue signatures as associated with functional divergence of the protein class. As mentioned above, our focus is on obtaining an initial, suboptimal hierarchy that is comparable to current CDD curated hierarchies and that can serve as a starting point for further optimization using either manual or automated methods. Here we describe statistically-based heuristic procedures that, in conjunction with Bayesian sampling, can obtain such an initial hierarchy from a multiple sequence alignment.

### Bayesian sampling over contrast alignment models

**Figure 1:**
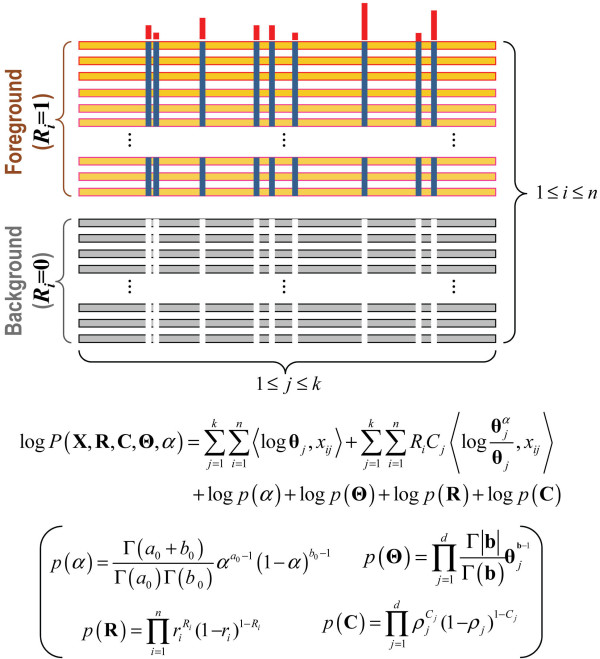
**Schematic drawing of a contrast alignment and the corresponding probability model.** Aligned sequences are assigned to either a ‘foreground’ or a ‘background’ partition (orange and gray horizontal bars, respectively). Partitioning is based on the conservation of foreground residues (blue vertical bars) that diverge from (or contrast with) the background residues at those positions (white vertical bars). Red vertical bar heights quantify the selective pressure imposed on divergent residue positions. Below this is given the logarithm of the corresponding probability distribution for the possible sequence partitions and corresponding discriminating patterns which together serve as the random variables over which sampling occurs. **X** is an *n × k* matrix representing a multiple alignment of *n* sequences and *k* columns; *x*_*i j*_ is a 20-dimensional vector of all 0’s except for a lone ‘1’ indicating the observed residue type; **R** is a vector indicating which rows (i.e., sequences) belong to the foreground (*R*_*i*_=1) or background (R_*i*_ = 0) partitions; **C** is a vector indicating which columns do (C_*j*_ =1) or do not (*C*_*j*_ =0) differentiate the foreground from the background; **Θ** is an array of vectors representing the amino acid compositions at each column position for each partition; $\langle \cdot ,\cdot \rangle$denotes the inner product of two vectors; and $\theta_{j}^{\alpha }\equiv \left(1-\alpha \right)\theta_{j}+\alpha \delta_{A_{j}}$ models the foreground composition at pattern positions where $\theta_{j}\equiv {\left(\theta_{j,1},\ldots ,\theta_{j,20}\right)}^{T}$ is the background amino acid frequency vector for column *j*, the parameter α specifies the expected background ‘contamination’ at pattern positions in the foreground, and δ_*Aj*_ is a vector that specifies the pattern residues at position *j*. At non-pattern positions, the vector *θ*_*j*_ corresponds to the overall (foreground and background) composition. The third through sixth terms in the equation correspond to the logarithm of the product of the prior probabilities with *p*(α) and *p*(**Θ)** defined by the beta and product Dirichlet distributions, respectively, and with *p*(**R**) and *p*(**C**) defined by independent Bernoulli distributions; prior definitions are as shown (in parentheses). The log-likelihood ratio (LLR) is computed by subtracting from the log-probability for the observed contrast alignment the log-probability for a ‘null’ contrast alignment, in which all of the sequences are assigned to the background partition.

Our approach relies on Bayesian Markov chain Monte Carlo (MCMC) sampling [39], which starts with an arbitrary model having a certain (conditional) probability that it (as opposed to other models) could have generated the input sequence alignment data. Then, in successive iterative steps, a number of alternative values for a given parameter of the current model are evaluated (while other parameters are held constant). A value for this parameter is then sampled proportional to its probability. This iterative process continues until convergence on the most likely models. Here we optimize in this way contrast alignment models, each of which consists of a pattern and a set of labels assigning each sequence to either a foreground partition or a background partition corresponding to sequences that either generally conserve or fail to conserve the pattern, respectively. A schematic representation of a contrast alignment and the corresponding probability distribution are described in Figure 1. To sample alternative contrast alignment models we use a MCMC sampling strategy [39], termed Bayesian Partitioning with Pattern Selection (BPPS) [40,41]. (MCMC sampling is required because, a priori, we know neither which sequences belong to the foreground, nor which positions are pattern positions, nor which residues are conserved at each pattern position.) The sampler converges on a model where the pattern best distinguishes the foreground from the background sequences.

### Multiple category functional divergence models

More recently, a multiple category (mc)BPPS sampler was developed [42] with a view to optimally assigning aligned sequences to various nodes within a predefined protein domain hierarchy based on functionally-divergent residue signatures. Thus the mcBPPS sampler aims to precisely define both the sequences belonging to each subgroup and the patterns most distinctive of each subgroup within a specific protein class. However, because the mcBPPS sampler does not define the hierarchy of contrast alignments, it requires that the user provide (as input) both a functional divergence (FD)-table (formally termed a “hyperpartition”) and seed sequences for each divergent subgroup. (Seed sequences serve as Bayesian priors or—if viewed as a missing data problem [43]—as labeled sequences that are required to remain in their pre-assigned subgroups during sampling and that thus help define each subgroup. The remaining (unlabeled) sequences are assigned to subgroups through Bayesian inference).

**Figure 2:**
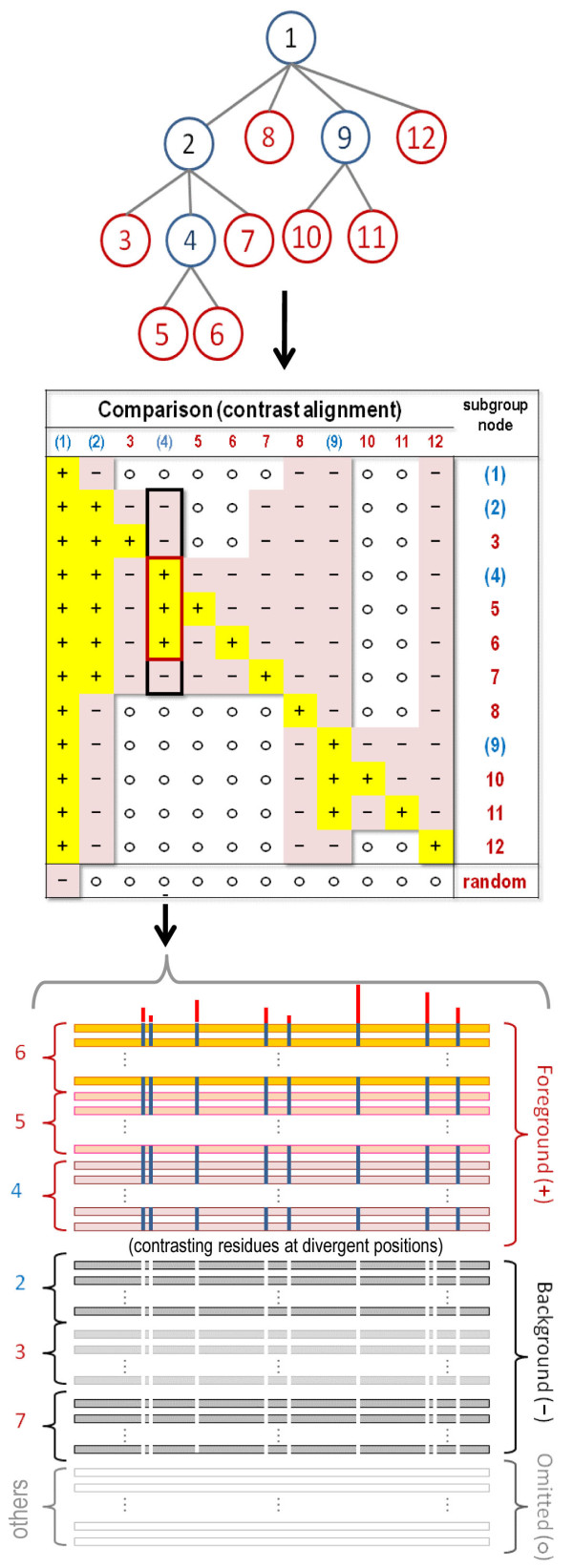
**A multiple category model optimized by the mcBPPS sampler.(top)** A tree representing the hierarchical relationships between functionally-divergent protein subgroups. Color code: internal nodes, blue; leaf nodes, red. Each subtree within the tree (i.e., each node and its descendents) corresponds to a set of sequences that generally conserve a pattern that sequences in the rest of the tree generally lack. For example, node 5 could represent a subfamily whose family, superfamily and class are represented by the subtrees rooted at nodes 4, 2 and 1, respectively. **(middle)** The corresponding functional divergence (FD-)table. A tree is converted into a FD-table, as follows: The subtree rooted at each node of the tree corresponds to the foreground (‘+’ rows) for that column in the table, whereas the rest of the subtree rooted at the parent of that node corresponds to the background (‘-‘rows). (A set of randomly-generated sequences serves as the background for the root node.) Each internal node in the tree corresponds to a miscellaneous category—that is to sequences sharing a common pattern with, but lacking patterns specific to each of its descendent subtrees. **(bottom)** Contrast alignment corresponding to column 4 of the table. Each subgroup corresponding to a row with a ‘+’ or a ‘-‘symbol in that column is assigned to the foreground or background, respectively; subgroups with an ‘o’ symbol are omitted from that contrast alignment.

Each row of a FD-table corresponds to a distinct functionally divergent subgroup of the input sequences and each column corresponds to a distinct contrast alignment whose foreground and background partitions are specified by the symbols in the table. Such a table is shown in Figure 2, which also illustrates the correspondence between a tree representing the hierarchical relationships between functionally divergent subgroups and the FD-table and between a column in the table and the contrast alignment; these are shown above and below the table, respectively. Given the relationships specified by the FD-table, the mcBPPS sampler stochastically reassigns aligned sequences to alternative subgroups and alternative patterns to each foreground partition until convergence on an optimal (or nearly optimal) set of contrast alignments. Modeling the functional divergence of an entire protein class in this way is substantially more powerful than using a single contrast alignment because: (i) In principle, it can optimally model every (functionally) divergent subgroup within an entire protein class concurrently. (ii) It sets up a stringent competition between functionally-divergent categories for pattern residues, thereby defining each pattern and partition much more precisely. (iii) It eliminates problematic sequences, which would otherwise tend to obscure analyses, by modeling them explicitly. Problematic sequences include, for example, related proteins that have further functionally diverged to become outliers, pseudogene products and other non-functional proteins, and unrelated or erroneous sequences. And (iv), by defining multiple categories of pattern residues within individual proteins it can reveal, in the light of available structural information, functionally important residue interactions. (For a mathematical description, evaluation and application of the mcBPPS sampler, see [42,44]).

Here we describe and apply an automated multiple category (amc)BPPS program that generates its own FD-table and seed sequences automatically and therefore merely requires a multiple sequence alignment as input. The number and nature of the partitions and the patterns is completely determined by the program. When used in conjunction with procedures for viewing structural interactions involving pattern residues, the amcBPPS sampler automates and enhances the creation and annotation of CDD hierarchical alignments. And, when linked into web-based BLAST searches, this can make previously inaccessible molecular information widely available.

## Results and discussion

In this section, we lay out the basic amcBPPS algorithm, illustrate an implementation of the algorithm as applied to P-loop GTPases, compare its performance against various manually-curated CD hierarchies, further evaluate its performance using both delete-half jackknifing and simulations, and apply it to several large protein classes for which existing hierarchies or alignments are currently unavailable.

### Algorithm

**Figure 3:**
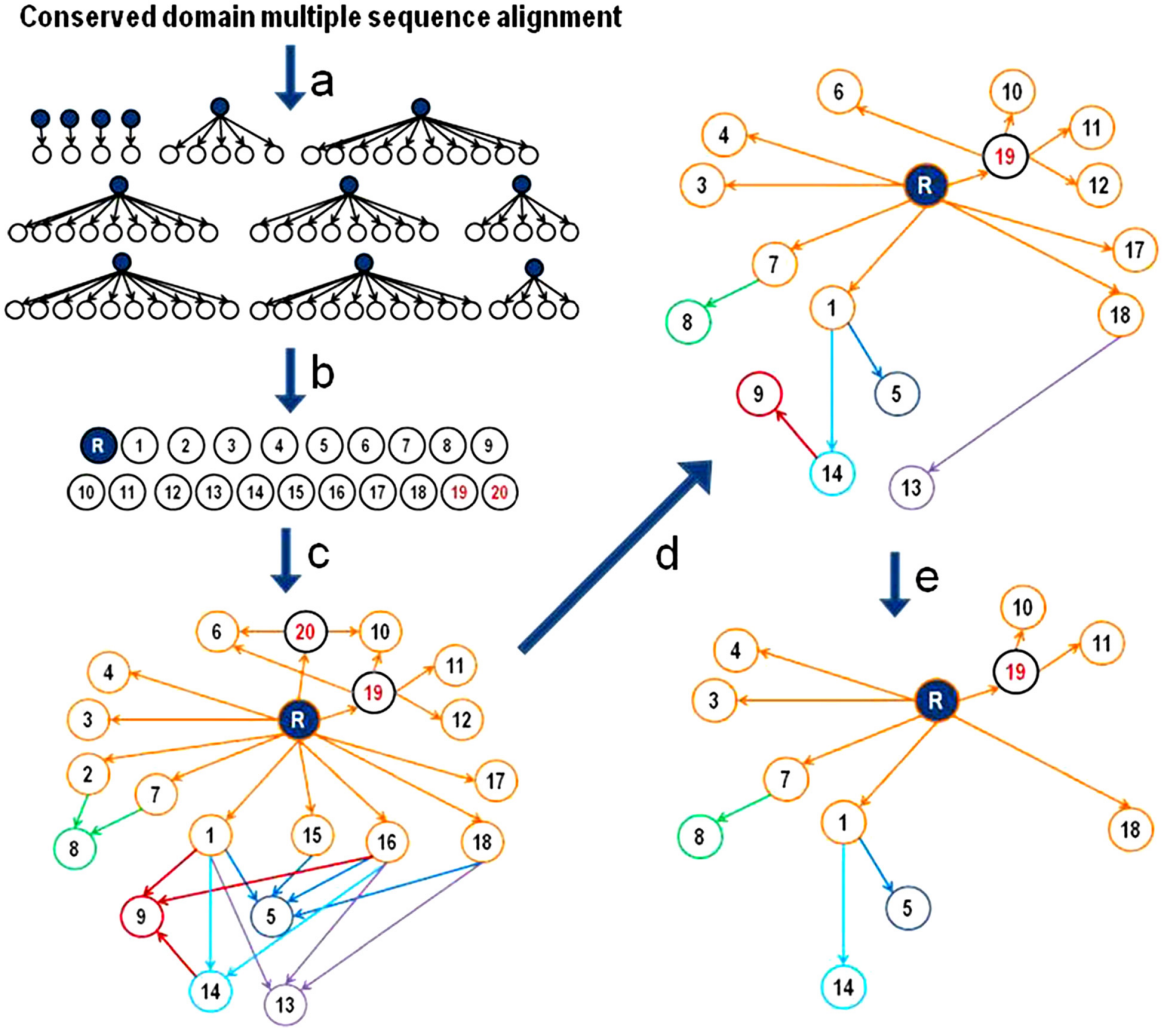
**The amcBPPS procedural substeps used to obtain a hierarchy from a multiple alignment.** Starting from a multiple sequence alignment for a particular protein domain, the amcBPPS program applies the following substeps (‘a’ to ‘e’) to create a domain hierarchy. Note that substep (a) corresponds to Step 1 of the amcBPPS algorithm whereas the other substeps correspond to Step 2. (**a**) Use heuristic procedures to create distinct FD-tables, corresponding to a forest of simple (rooted, branchless) trees; each leaf of a given tree corresponds to a distinct subgroup within the protein class. (The mcBPPS sampler is used to optimally assign sequences to each leaf node; different prior probability settings can be used to favor convergence on subfamilies, families or superfamilies.) (**b**) Select leaf nodes from the forest corresponding to more or less distinct, functionally divergent subgroups; this is done by combining each set of nearly identical nodes into a single set. Define a root node (labeled R in the figure) corresponding to the universal sequence set. Larger superfamily nodes (labeled with red integers) also are created from related leaf nodes. The haze around nodes indicate the partially-overlapping nature (i.e., fuzziness) of the corresponding sequence sets. (**c**) Generate a directed acyclic graph (DAG) representing superset-to-subset relationships between nodes and with arcs weighted by (the negative of) the corresponding log-likelihood ratios (LLRs) associated with the BPPS statistical model. For clarity, nodes and arcs directly connected to the root are shown in orange whereas other (non-root) nodes are uniquely colored. (**d**) Obtain from the DAG a shortest path spanning tree using a breadth-first scanning algorithm [45]. Because the arcs are weighted using LLRs, this procedures returns a maximum likelihood tree associated with the DAG. (**e**) Prune nodes that both are directly attached to the root and significantly overlap with other nodes and thus correspond to ill-defined sequence sets. For the remaining nodes, remove the overlap between their corresponding sequence sets (see text for details) and prune from the tree those nodes that lack a minimum number of sequences (30 by default). This typically yields a reduced hierarchy (as shown), which is converted into a FD-table (as illustrated in Figure 2) for optimization by the mcBPPS sampler.

The amcBPPS algorithm aims to identify the hierarchical relationships between functionally-divergent subgroups within an entire protein domain class based on the differentiating patterns present in that class. It does this by defining: (i) the number of sequence sets, (ii) the members of each set, (iii) the hierarchical relationships between sets and (iv) the corresponding functionally divergent patterns. This is accomplished in three steps. Steps 1 and 2 constitute the novel aspect of the program by providing input to the mcBPPS sampler in Step 3; these first two steps also speed up convergence in Step 3 by providing a better starting point for the mcBPPS sampler (the algorithmic details of which are described in [40,42]). Conceptual aspects of the algorithm corresponding to Steps 1 and 2 are illustrated in Figure 3 (algorithmic details are provided as pseudocode within Methods). These first two steps are performed heuristically and thus constitute an informed guess on how to best model the protein class (based on the same statistical criteria used in Step 3). The sampler then improves upon this model by optimizing sequence and pattern assignments within this hierarchy. The hierarchy returned by the amcBPPS program may be edited (and thus further refined) and then optimized again by the mcBPPS program. Such editing may, for example, further subcategorize previously identified or miscellaneous subgroups.

### Identifying simple subgroups (Step 1)

Step 1 of the amcBPPS algorithm (represented by the arrows labeled ‘a’ and ‘b’ in Figure 3) first generates a forest of simple (rooted and branchless) trees, the leaves of which correspond both to functionally-divergent subgroups within the protein domain class and to sub-alignments of the input alignment. This is accomplished by obtaining seed sequences and using them to create these trees, as follows: (i) All closely-related sequences (by default, those sharing ≥ 95% identity) from the same phylum are clustered into a common set (that are thus disjoint from other such sets). (ii) All pairs of moderately related sequences (by default, those sharing ≥ 40% identity) from distinct phyla are stored on a heap (also called a priority queue) using their pairwise scores as the key. (iii) Iteratively remove the top-scoring cross-phylum pair from the heap and merge their two disjoint sets into one set. (Merging is done using an efficient algorithm described by Tarjan [45]). (iv) Once a disjoint set contains sequences from a pre-defined minimum number of distinct phyla (four, by default), the sequences of highest rank from each phylum are used to seed a new subgroup; the disjoint set is then labeled to avoid picking this subgroup repeatedly. (v) Keep generating subgroups in this way until a pre-defined number of seed sequence sets are obtained (typically 1–10), at which point a simple FD-table is constructed where each subgroup node is a direct descendent of the root node. (For the correspondence between a FD-table and the nodes of a tree, see Figure 2). And (vi) repeat substeps iii-v until all sequence pairs have been removed from the heap. To ensure that the subgroups are sufficiently diverse, we require that each seed set consensus sequence share less than a specified level of sequence identity with other seed set consensus sequences (< 40% identity, by default). Taken together, these sub-steps favor the identification of the most conserved and phylogenetically diverse subgroups.

For each of these FD-tables (and the corresponding seed sequences) the mcBPPS sampler assigns each of the multiply aligned input sequences to a subgroup (as specified by the rows in the table) and determines the differentiating conserved pattern for each contrast alignment (as specified by the columns in the table). To ensure that subgroups at different levels of the hierarchy are identified, the algorithm performs multiple runs using various numbers of leaf nodes and various prior probability settings for *P*(R), *P*(α) and *P*(C) (which are defined in Figure 1). Convergence on protein subfamilies is favored by specifying a high number of leaf nodes (by default 10), by lowering the (Bernoulli distributed) prior probability for assigning a sequence to a leaf node, *P*(R), (where by default, *r*_*l*_ = 0.01), by setting the (beta distributed) prior probability, *P*(α), to favor a lower degree of background contamination by assigning more pseudo-observations to pattern matching residues and fewer pseudo-observations to contaminating residues (by default, *a*_*0*_ = 9 and *b*_*0*_ = 1) and by raising the (beta distributed) prior probability that a column corresponds to a pattern position, *P*(C) (by default, *ρ*_*j*_ = 0.01). The rationale for choosing these settings is that, for subfamilies, membership is more exclusive, sequences are more highly conserved and, consequently, conserved patterns more extensive. (Note, however, that, in the absence of such a rationale, non-informative priors are used by default (e.g., uniform beta and Dirichlet distributions) in order to maximize the influence of the data on model optimization.) Convergence on a super-family is favored by specifying a single subgroup and by altering these prior parameter settings accordingly (where by default, *r*_*l*_ = 0.2, *a*_*0*_ = 1, *b*_*0*_ = 1 and, *ρ*_*j*_ = 0.0001). Default settings are based on applications to actual protein sequences, though it should be noted that the influence of these prior settings is minor. Hence these priors primarily function as tuning parameters to help gently guide the sampler into finding a variety of functionally divergent subgroups. To avoid finding the same subgroup repeatedly, sequences assigned to a subgroup in a previous run are prohibited from being used as seeds in subsequent runs. Subfamilies can also be identified recursively; that is, by rerunning the program on a single subgroup in order to find subgroups within subgroups (though this approach is not used here). The pseudocode for this step of the amcBPPS algorithm is given in Methods.

### Defining a hierarchy for the protein class (Step 2)

Once individual subgroup sets are identified in Step 1 (see arrow labeled ‘b’ in Figure 3), the program hierarchically arranges these into a more complex tree, from which a FD-table is obtained. It does this using efficient bitwise set operations [46], standard network algorithms [45] and pattern-based statistical criteria, namely the contrast alignment log-likelihood ratio (LLR) used by the mcBPPS sampler [40,42] (the basic component of which is given in Figure 1). These hierarchically-arranged subgroup sets are ‘fuzzy’ due to the uncertainty associated with set membership (being based, as it is, on imperfectly conserved discriminating patterns). Thus Step 2 of the algorithm (which corresponds to the arrows labeled ‘c’ through ‘e’ in Figure 3) determines which of the input sets (from Step 1): (i) are the same set; (ii) are distinct sets; or (iii) are supersets of another set or sets. Step 2 is subdivided into three sub-steps: (2a) merge each collection of subgroup sets deemed to be identical into a single set; (2b) cluster related subgroup sets into common supersets; and (2c) create a tree representation of the subgroup hierarchy, which is done using a breadth-first scanning algorithm [47] to find a shortest path tree [45]. Step 2c also refines the tree to eliminate inappropriate overlap between sets while also eliminating nodes from the tree that, as a result of this refinement process, are no longer statistically significant. The pseudocode for Step 2 is given in Methods. From this tree a FD-table is then generated as input to Step 3.

### The mcBPPS sampler (Step 3) and further refinements

The output from Step 2 provides a starting point for mcBPPS sampling, which optimizes the patterns and partitions corresponding to the FD-table. The basic statistical and algorithmic aspects of the mcBPPS sampler were previously described [42]. To further expand a CD hierarchy the output files obtained from an initial amcBPPS analysis can also be used to recursively analyze, in the same way, several of the larger subgroups. To do this, the output alignment file for a major subgroup is used as an input file for the amcBPPS program. Likewise, a CD hierarchy can also be refined by editing the FD-table manually and then applying the mcBPPS sampler, as was previously described [42]. To speed up analysis of a given subtree, such manually edited FD-tables (guided by the tables obtained automatically) may be designed to expand subgroups within that subtree, while modeling the other branches of the hierarchy only at the highest levels (e.g., by modeling other subtrees off the main root as single nodes). In keeping with the automated theme of this article, however, we will not describe in detail nor apply these approaches here.

### Implementation and testing

The amcBPPS algorithm was implemented in C++ (executables are available from the corresponding author), applied to various protein classes and the output compared to manually-curated CDD alignment hierarchies (when available). A wide range of CDD hierarchies—from preliminary to well-developed releases (as well as some out-of-date versions)—were examined in this way. Input multiple alignments were obtained by using the NCBI hierarchy of CD alignments as input to the MAPGAPS program [48], which detected and aligned related protein sequences within the NCBI nr, env_nr and translated EST protein databases. To obtain input alignments corresponding to large protein classes for which CDD hierarchies are not yet available we used alternative procedures, as described in Methods.

**Figure 4:**
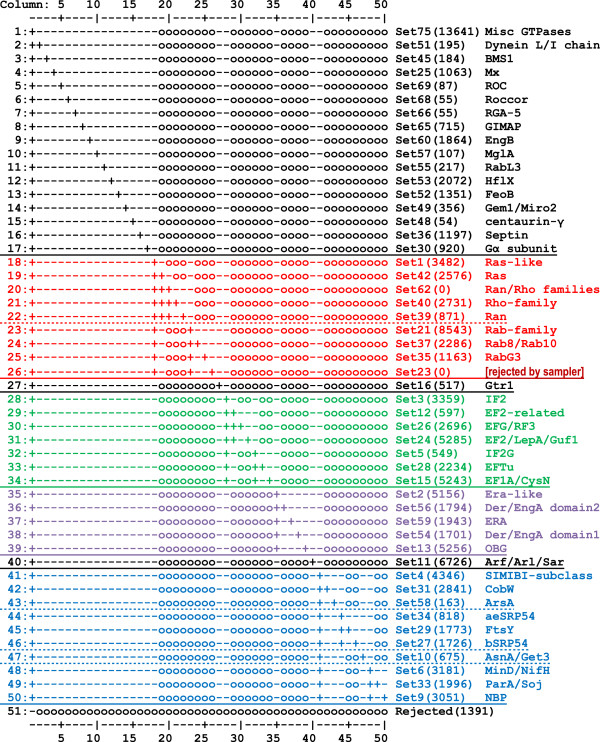
**FD-table for P-loop GTPases.** The number of sequences in each subgroup are given in parentheses. Major subtrees are color coded.

Illustrative example: P-loop GTPases. To familiarize the reader we begin by illustrating our approach with an analysis of P-loop GTPases. Using an input alignment of 198,624 P-loop GTPases, the amcBPPS program returned the FD-table shown in Figure 4. (To make Figure 4 more readable, this was performed using parameter settings that favor a smaller hierarchy than was found for Table 1). It also returns a corresponding set of contrast alignments, which highlight the pattern residues identified by the sampler; one such alignment is shown in (Additional File 1: Figure S1). Note that the sampler will reject heuristically proposed subgroups whose existence is not supported by the data (such as Set23 in row 26 of Figure 4). Further subdivision of the hierarchy in Figure 4 may be accomplished by recursively applying the amcBPPS sampler to a previously-identified subgroup. (Additional File 1: Figure S2) illustrates this for the Ras-like GTPases by showing an expanded subtree corresponding to the column 18 foreground partition in Figure 4. By applying the amcBPPS sampler recursively in this way, a very extensive hierarchy may be obtained.

**Table 1:** Comparison of curated and automatically-generated domain hierarchies

**CDD**	**Protein superfamily**	**number**	**length**	**Manually curated**		**Automatically generated**		
**Ident.**		**seqs**^**‡**^		nodes^*^	**LLR**^**†**^	nodes^*^	**LLR**^**†**^	**time**^**§**^
**cd00030**	C2	23,452	102	106 (103)	236574	78(73)	223857	19.4
**cd00138**	PLDc_SF	16,765	119	105 (102)	241766	36(34)	192876	10.0
**cd00142**	PI3Kc_like	2,409	219	22	34129	16	34563	4.5
**cd00159**	RhoGAP	4,815	169	39(38)	55604	32	53540	7.97
**cd00173**	SH2	5,917	79	111 (101)	49274	39	40075	3.5
**cd00180**	Protein kinases	104,912	215	280(260)	1378273	107(104)	1536991	241.0
**cd00229**	SG NH_hydrolase	14,635	187	30	180667	29	183822	14.95
**cd00306**	S8/S53 peptidase	10,960	241	36	161685	45(44)	173693	30.90
**cd00368**	Molybdopterin-Binding	9,540	374	26	177569	44	209704	39.3
**cd00397**	DNA_BRE_C	25,824	164	27 (26)	187382	39(37)	211739	16.9
**cd00761**	Glycosyltransferase A (GT-A)	66,260	156	71 (70)	944727	123(110)	1048396	193.8
**cd00768**	Class II aaRS-like core	37,160	211	17	674454	31	833691	54.3
**cd00838**	MPP_superfamily	33,753	131	61	402297	55(54)	399553	65.1
**cd00900**	PH-like	22,593	99	81	211812	99(98)	274945	52.3
**cd01067**	Globin_like	9,933	117	4 (1)	11133	26 (25)	73808	4.3
**cd01391**	Periplasmic_Binding_Protein_1	36,330	269	142(140)	619713	68(65)	580753	169.1
**cd01494**	AAT_I (Pyrodoxal-PO4-binding)	114,781	170	16	1086328	92(84)	2027660	249.67
**cd01635**	Glycosyltransferase GTB	44,366	229	45	723443	95(93)	881414	232.7
**cd02156**	Class I aaRS-like core √	53,605	105	34	522962	61(57)	698273	41.4
**cd02883**	Nudix_Hydrolase	32,046	123	55 (54)	321636	61(60)	367819	43.2
**cd03128**	GAT-1 (mcBPPS vs pmcBPPS)	46,514	92	34(32)	319515	64(62)	388621	42.2
**cd03440**	hot_dog	30,162	100	22(18)	141990	70 (69)	345298	39.1
**cd03873**	Zinc peptidases	24,455	237	81	596408	69(66)	590521	43.9
**cd05466**	Periplasmic_Binding_Protein_2	45,287	197	76(73)	523941	49(41)	411445	31.7
**cd06587**	Glo_EDI_BRP_like	36,165	112	60 (58)	335848	94(91)	479522	54.8
**cd06663**	Biotinyl-lipoyl	25,013	73	4	53038	25(18)	66571	4.53
**cd06846**	Adenylation_DNA_ligase_like	3,833	182	14	43276	20	48,475	4.8
**cd08555**	PI-PLCc_GDPD_SF	8,707	179	74 (73)	143201	37(32)	123075	6.9
**cd08772**	GH43_62_32_68 (β propellers)	6,760	286	28	111336	51(50)	176701	30.0
**cl09931**	Rossmann fold proteins	424,764	93	361 (347)	4110907	145(130)	4029120	757.2
	**Average**	44,057	167.7	66.4	486696	56.9	556884	83.6

^**‡**^ After removing identical sequences and sequences that fail to align with at least 75% of the domain.

^*^ Numbers in parentheses indicate the nodes retained after insignificant nodes were removed by the mcBPPS program.

^**†**^ The log-likelihood ratio in nats.

^**§**^ The time (in minutes) is for Steps 2 and 3 of the algorithm only; Step 1 can be parallelized to run in less than 10% of the time shown.

### Criteria for comparing hierarchies

To assess how well the amcBPPS program performs relative to curated CD hierarchies, we compared its output against 30 manually curated CDD hierarchies (Table 1). Before considering this analysis, however, we first need to discuss the criteria used to evaluate and compare hierarchies.

### Lack of gold standards

CDD hierarchies have been carefully constructed by expert curators and therefore come the closest to a benchmark set for evaluating the amcBPPS sampler. However, as this study reveals, certain aspects of CDD hierarchies lack statistical support or are incomplete or incorrect for various reasons: For example, CDD hierarchies are typically at different stages in an ongoing refinement process, and, for protein domain classes consisting of tens or hundreds of thousands of sequences, the number of possible hierarchies to consider is astronomical, which makes optimization through manual curation extremely difficult. Furthermore, due to the stochastic nature of and the inability to directly observe evolutionary divergence, it is impossible to eliminate the inherent uncertainties associated with protein classification. Hence, for the present study our aim is merely to replicate the current manual curation process by generating hierarchies of comparable quality automatically, thereby dramatically speeding up the current labor-intensive curation process.

### Comparison criteria for this analysis

**Figure 5:**
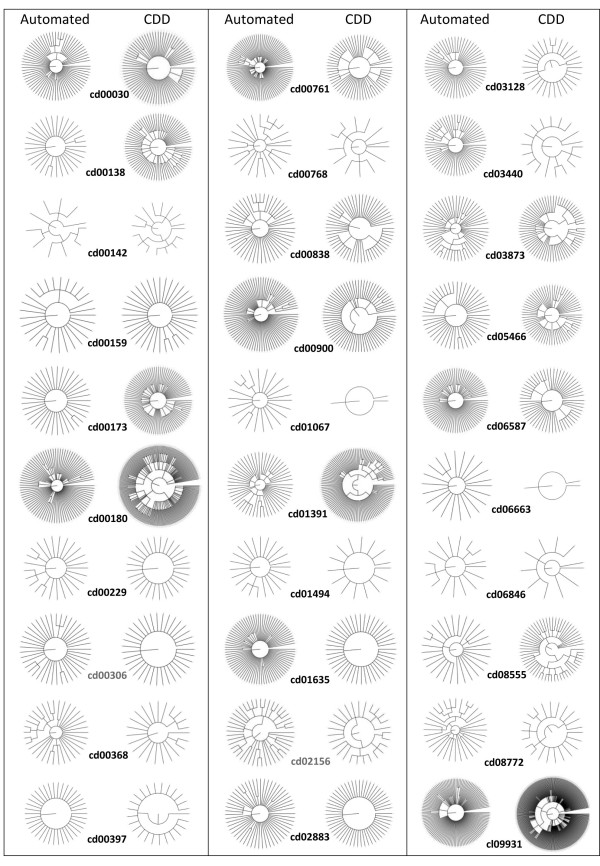
**Comparison of curated and automatically-generated hierarchies.** Hierarchies are shown as circular trees.

Despite the absence of a gold standard, the statistical criteria used by the amcBPPS program provide a way to compare two hierarchies for the same conserved domain. It does this by determining objectively whether or not (and, if so, to what degree) the sequences in each protein subgroup have diverged from the evolutionarily related subgroups indicated by a specific hierarchy. This measure is expressed as a log-likelihood ratio (LLR), where non-positive values indicate a lack of statistical support for a functionally divergent event within the hierarchy. Such a comparison is performed as follows: We are given two heuristic methods for obtaining a (presumably suboptimal) hierarchy: one manual and one automatic. To compare the two methods, we first use each hierarchy (along with a corresponding multiple sequence alignment) as input to the mcBPPS sampler, which then optimizes the patterns and sequence partitions associated with that hierarchy and returns an optimized log-likelihood ratio (LLR). Because this optimizes the automatically-generated and manually-curated hierarchies in the same way based on the same statistical criteria, the only difference is that the hierarchies and seed alignments were obtained either automatically or through manual curation. Thus, by comparing their optimized LLR scores, we can obtain a measure of the relative performance of the two methods. In addition, we also determine the degree of overlap between the two hierarchies as a qualitative indication of the similarity of the two hierarchies. The results of such comparisons are summarized in Table 1 and Figure 5.

### Evaluation of the amcBPPS program

To evaluate the amcBPPS program over a wide variety of input, we chose the 30 conserved domains given in Table 1. These domains vary in the numbers of members detected in the protein databases (from a few thousands to hundreds of thousands of sequences as indicated in column 3), in the lengths of their conserved core (from 73 to 374 residues; column 4), and in the size (column 5) and complexity (Figure 5) of their curated hierarchies.

### Comparisons with manually curated CDD hierarchies

Based on the LLR statistic the automatically-generated hierarchies (column 8) are comparable to the corresponding manually-curated hierarchies (column 6) and are, in fact, slightly better on average (556,884 nats versus 486,696 nats for the curated hierarchies). Manual and amcBPPS hierarchies (Figure 5) were also compared qualitatively by determining the degree to which the sequence sets corresponding to the nodes in one hierarchy overlap with those of the other hierarchy; in Additional File 1: Figure S3 illustrates such a comparison for the PI3Kc_like domain hierarchy (cd00142). This provides a detailed comparison of two hierarchies by computing how the sequences at each level of one hierarchy are assigned to each level of the other hierarchy and vice versa. Such comparisons indicate that the differences between the curated and amcBPPS hierarchies are mainly due to the following: (i) A node in one hierarchy being modeled as a subtree in the other. (ii) Additional child nodes being added to parent nodes in one hierarchy but not the other. (iii) A subtree in one hierarchy being split into unrelated subtrees (or nodes) in the other due to a failure to join these to a common internal (parent) node. And (iv) inherently ambiguous sequences that can’t be clearly assigned to a specific node in the hierarchy; such sequences may correspond to pseudogene products or functionally defective members of a protein class that are difficult to categorize because they harbor degenerate subgroup patterns. Of course, the larger and more functionally divergent hierarchies are more challenging.

Most of the differences between the manual and automated hierarchies are due to fundamental differences between the two approaches (as revealed by examination of comparative analyses like the one shown in Additional File 1: Figure S3). Curated hierarchies may rely, in part, on information that is (currently) ignored by the amcBPPS program, such as subfamily-conserved inserts and 3D structures—though, on the other hand, the amcBPPS program utilizes a far greater amount of sequence data that is also up-to-date. In contrast, some of the CDD heirarchies may be out-of-date or still incomplete. The amcBPPS program also requires each node to (initially) correspond to at least 30 sequences (by default) in order to avoid statistical biases due to small sample size. CDD curators, however, may construct subgroups containing fewer sequences. Likewise, the amcBPPS program selects seed sequences from at least three or four distinct phyla in order to avoid sampling biases introduced by orthologous sequences from closely related organisms. Hence, it will fail to identify subgroups that only occur in vertebrates, for instance. (Of course, this restriction could be relaxed somewhat by using less conservative, yet still valid criteria.) In contrast, CDD curators may choose representative sequences (which were used as seeds for the analyses in Table 1) from more closely related taxa. Due to such restrictions, an amcBPPS-generated hierarchy tends to have fewer nodes (i.e., rows in the FD-table) because it is prohibited from identifying certain CDD-defined subgroups. This also tends to lower the LLR, which (other things being equal) increases with the number of nodes in the hierarchy. (This increase occurs at a slower rate as the number of nodes increases, however, inasmuch as the most strikingly divergent subgroups are typically modeled first.) Despite these differences, after taking these considerations into account, we found the amcBPPS hierarchies to be persuasively consistent with the corresponding CDD hierarchies (Table 1). A perceived “unfair” advantage of the amcBPPS algorithm might be that it utilizes the same statistical model to construct a hierarchy that is used to score that hierarchy, whereas curators do not. However, subsequent (pattern-partition) optimization of both hierarchies using the mcBPPS sampler should counteract this advantage. That is, assuming that the curated hierarchy is in fact superior and that our statistical model is biologically meaningful, then optimal partitioning of the sequences and optimal pattern assignment by the sampler for both types of hierarchies should result in a superior LLR score for the curated hierarchy.

**Figure 6:**
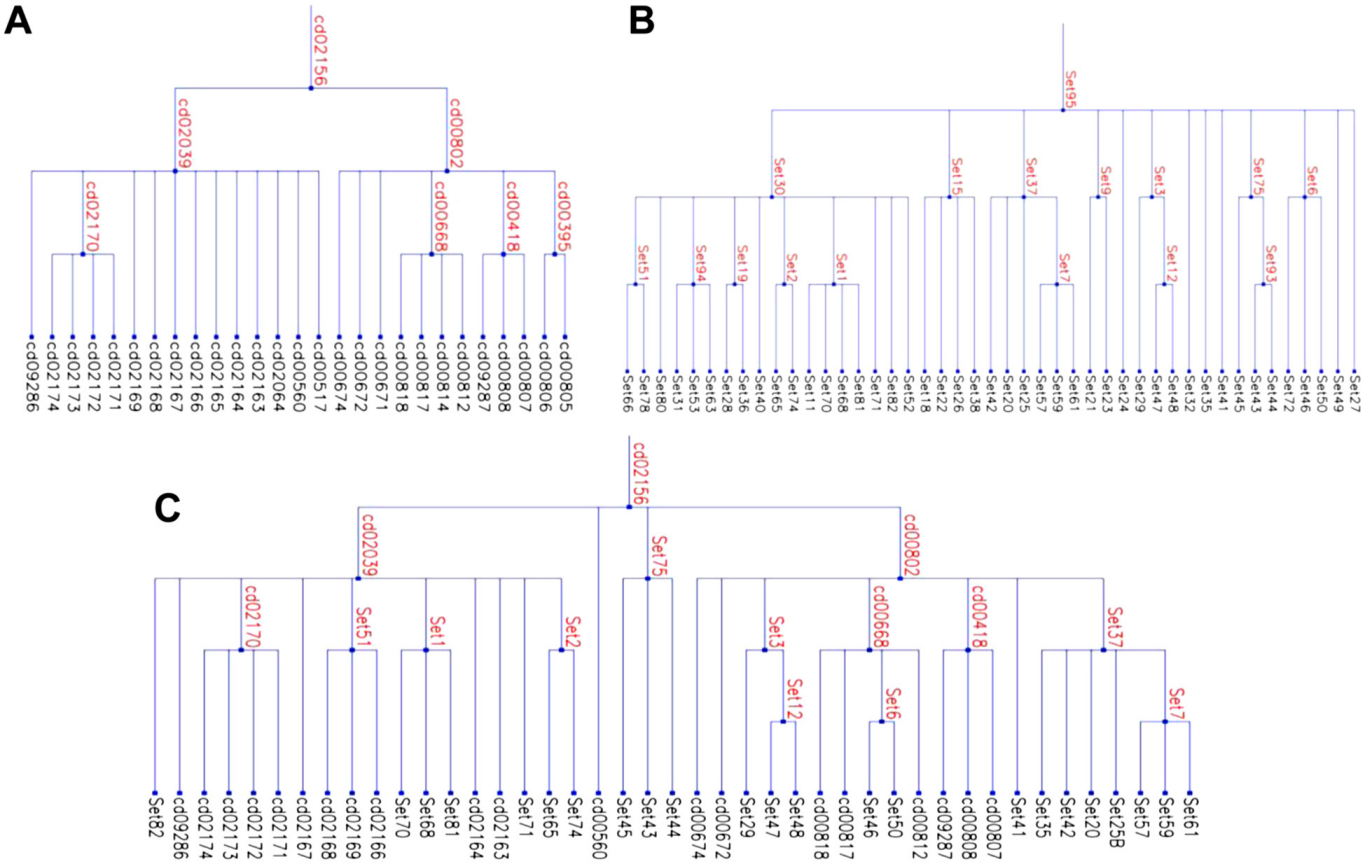
**Improving a hierarchy by merging features of curated and amcBPPS hierarchies.** Shown are hierarchies for cd02156 in Table 1. (**A**) The original CDD hierarchy. (**B**) The automatically generated hierarchy. (**C**) A hybrid hierarchy created by incorporating features of both (**A**) and (**B**).

Unsurprisingly, our analysis also indicates that the hierarchies obtained both manually and automatically are typically suboptimal. For example, manual and amcBPPS hierarchies for the S8/S53 peptidase domain (cd00306) had LLRs of 161,685 and 173,693 nats, respectively, whereas a hybrid hierarchy containing features of both of these has a LLR of 177,727 nats. Figure 6 likewise illustrates the construction of a hybrid hierarchy for the class I aaRS-like core domain (cd02156), which improves the LLR from 522,962 (for the CDD hierarchy) to 652,987 nats. Examining LLR statistics can suggest other ways in which to improve CDD hierarchies. For example, within the Glo_EDI_BRP_like hierarchy (cd06587 in Table 1), the mcBPPS sampler rejected an intermediate node (cd07240) to which it had assigned a LLR of −433 nats. An investigation to determine why this occurred revealed that, based as well on the criteria used by the CDD curators, the cd07240 intermediate node is incompatible with several of its leaf nodes, which are therefore better modeled as direct descendents of the root node. For these and other domains in Table 1, suggested improvements in CDD hierarchies based on LLRs were corroborated through manual inspection by the CDD resource group.

### Delete-half jackknife analyses

A bootstrap or jackknife [49] procedure can be used to estimate confidence levels for evolutionary trees [50]. However, applying this approach to a CD hierarchy is complicated by the potential run-to-run variability both in the number of the leaf nodes and in the associated sequence sets. Thus existing evolutionary tree bootstrap and jackknife procedures, which require that each of the sampled trees use the same set of leaf nodes, cannot be used. Instead, we implemented a delete-half jackknife procedure that—though unable to provide quantitative confidence levels for specific features of a hierarchy—can nevertheless provide a qualitative assessment of the run-to-run variability of amcBPPS-generated hierarchies. This involved running the amcBPPS program on each of 24 different input alignments (the domain identifiers of which are given in Methods) after randomly removing half of the sequences. Given ten such runs for each domain, we compared the consistency of the resultant hierarchies from run to run as follows: For each pair of runs (i.e., 45 pairs for each domain tested) we determined how those input sequences shared by both hierarchies (i.e., about one-fourth of the sequences in the input alignment) were partitioned among the nodes of one hierarchy relative to the other hierarchy. An example output file is shown in (Additional File 1: Figure S4).

For these analyses we found that, among the leaf node sets in one tree that share at least one sequence in common with a leaf node set in the other tree, on average 47% share precisely the same set of sequences (i.e., among those sequences present in both trees) and 74% share more than 90% of their sequences in common. Moreover, in most cases where an identical sequence set is not found, the missing sequences were typically assigned, not to unrelated leaf nodes, but either to a parent node further up the tree or to the rejected sequence set. Among the remaining cases, a node in one hierarchy is either split into multiple nodes or (in the worst case) split between nodes in the other hierarchy. At times a hierarchy could end up omitting certain nodes due to the delete-half jackknife procedure removing sequences belonging to certain phyla resulting in insufficient phylogenetic diversity to seed the formation of a subgroup. Of course the topologies (shapes) of the jackknife trees found by the sampler also differ, which is a common problem associated with evolutionary trees consisting of large numbers of distantly related sequences. This is presumably due in large part to the amcBPPS algorithm failing to find the optimal topology—an issue that, in the future, we will address by sampling over alternative topologies. Of course, both this future sampler and the jackknife procedure applied here will be useful for identifying the most reliable features of a hierarchy. Taken together, these results confirm the observation we made in the previous section, namely that the amcBPPS program generally finds a suboptimal hierarchy that, nevertheless, provides a good starting point both for curation and further automation. Output from these jackknife analyses are available at http://www.chain.umaryland.edu/amcbpps/jackknife.txt.

### Simulations

As an additional check, we implemented a procedure to generate simulated sequences from profile HMMs where each such profile corresponds to a node from one of the 24 domain hierarchies used in the jackknife analysis. The rationale for doing this was to determine how well the amcBPPS program identifies sequences corresponding to predefined subgroups. Note that this procedure captures sequence features of each subgroup, but not how those subgroups are hierarchically arranged. For each node of each hierarchy we generated the same number of aligned sequences as were assigned to that node in the original hierarchy. After running the amcBPPS program on each of these simulated alignments, we determined the degree to which each set of related simulated sequences were correctly modeled as belonging to a single subgroup. An example output file in (Additional File 1: Figure S5) illustrates how the amcBPPS program correctly categorized nearly all of these sequences given the structure of the inferred hierarchy (on average 69% of the sampled simulated sets correspond exactly to the HMM-generated sequence sets). Output from simulations for the 24 domains is available at http://www.chain.umaryland.edu/amcbpps/simulate.txt.

### Time complexity

The computationally most intensive routine in Step 1 of the amcBPPS program is an all-versus-all pairwise comparison of pre-aligned sequences (with indels ignored). This has a time complexity of O(*k*·*m*^2^) = O(*n·m*) where *k* is the number of aligned columns, *m* is the number of sequences and *n = k*·*m* is the effective size of the input alignment. In addition, Step 1 involves a simpler version of the Step 3 algorithm that, of course, exhibits the same time complexity as Step 3 (see below) as well as other operations that perform better than O(*n·m*) (e.g., heap and disjoint set operations on *m* sequences) [45]. Hence the time complexity for Step 1 is O(*n·m*).

**Figure 7:**
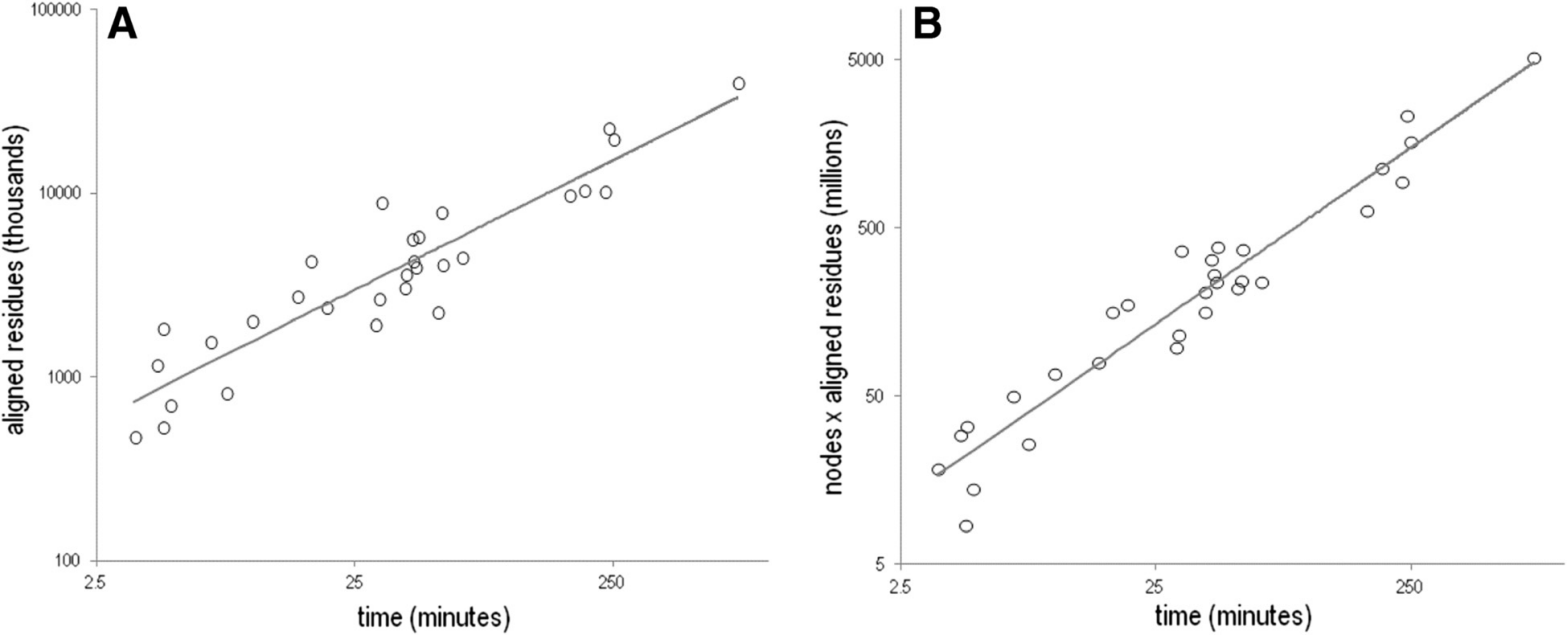
**Time complexity of Steps 2 and 3 of the amcBPPS program.** (**A**) Plot of run times versus the number of aligned residues in the input multiple alignment. Shown are data points from Table 1 and the corresponding linear regression trend line (*r* = 0.95). Because this plot is shown using a logarithmic scale for both axes, the observed time complexity O(n) of the program can be estimated from the slope of the trend line: Since time *t = c n*^*k*^, it follows that log*t* = logc + *k* log*n* on a log-log plot. The slope of the trend line is *k* = 1.2 indicating an observed time complexity somewhat worse than linear. (**B**) Plot of run times versus the number of aligned residues times the number of nodes in the hierarchy created in Step 2. This plot results in a slightly better fit (r = 0.98). The slope of the trend line is *k* = 0.9 indicating an observed time complexity that is essentially linear.

The time complexity of Steps 2–3 is unclear based on the underlying algorithm. Therefore, using a plot of the run times for the amcBPPS analyses in Table 1 versus the size of the input alignments, we estimate that the time required for Steps 2–3 scales as O(*n*^*1.2*^) (see Figure 7A). Assuming that the asymptotic time complexity for Steps 2–3 is indeed O(*n*^*1.2*^), which admittedly may not be the case given our empirically-based approach, then whether or not O(*n*^*1.2*^) is better than O(*n·m*) depends on the ratio of *k* to *m*^*4*^. (Step 1 and Steps 2–3 are asymptotically identical when *n·m* = *n*^1.2^ which implies that *k* = *m*^4^.) Step 1, which is O(*n·m*), performs asymptotically worse when *k* < *m*^4^ and Steps 2–3, which is O(*n*^*1.2*^), is worse when *k* > *m*^4^. Since for essentially all protein domains *k* < *m*^4^ the time complexity of the amcBPPS program (i.e., Steps 1–3) appears to be O(*n·m*). It is important to note, however, that Step 1 (which incidentally is easily parallelized) required less time than Steps 2–3 in our analyses—even on the largest input alignments—suggesting that constant factors rather than asymptotics are influencing program performance.

Because the run times for Steps 2–3 are also likely to depend on the size of the hierarchy generated by the program in Step 2, Figure 7B plots the run times versus the number of aligned residues times the number of nodes in the hierarchy (i.e., rows in the FD-table). This yields a slightly improved, essentially linear dependency. This observed time complexity is largely due to Step 3 being more or less independent of the number of nodes, which is achieved by computing conditional posterior probabilities (the most time consuming routine) for each column of the FD-table only when considering the assignment of a sequence to one of two possible new partitions rather than to one of a typically much larger number of rows. Thus the amcBPPS program can be applied to very large multiple sequence alignments, which is important given the current rapid increase in sequence data.

### Analysis of protein domains lacking CD hierarchies

There are a significant number of protein domains for which a CDD hierarchy has not yet been constructed. In some (though not all) cases a single curated alignment is available as a starting point. To test the performance of the amcBPPS program in such cases, we chose 10 domains, for which curated alignments were available, and two domains, for which we first constructed an alignment using Bayesian multiple alignment methods [51,52] (see Table 2). We then applied the MAPGAPS program to these alignments to obtain much larger multiple alignments as input to the amcBPPS program. The amcBPPS-generated hierarchies were then evaluated by mapping each node’s sequence subgroup onto a phylogenetic tree constructed for all sequences in the hierarchy. Such sequence alignment derived phylogenetic trees are used by CDD curators, both to get started on an initial subfamily classification and to iteratively refine that initial hierarchy (often over a period of weeks or months). This reveals that the amcBPPS-generated hierarchies agree very well with how the CDD curators would subgroup the sequences based on such a tree. Additional File 1: Figure S6 shows part of such a sequence tree computed from the input sequences used for RNA recognition motif domains (cd00590) with the amcBPPS hierarchy mapped onto the tree using a color coding scheme. This confirms that the amcBPPS program can substantially speed up the curation process when starting from scratch.

**Table 2:** Protein domain hierarchies generated automatically either from a single curated alignment or from non-aligned sequences

**identifier**	**Protein superfamily name**	**# seqs**	**# nodes amcBPPS**	**LLR**	**Run time**^**§**^
**Started from curated alignments:**					
**cd00075**	Histidine kinase-like ATPase c	87,258	95(62)	**518062**	119.27
**cd00130**	PAS	50,200	117(115)	416375	103.95
**cd00174**	SH3	13,890	44(35)	26971	3.83
**cd00590**	RRM	107,488	63(56)	557782	63.75
**cd01427**	HAD-like hydrolases	41,818	85(73)	324699	59.77
**cd02440**	AdoMet_MTases	150,872	112(99)	1417985	250.27
**cd04301**	NAT-SF	43,486	71	244420	23.30
**cl02566**	SET (pfam00856)	8,946	21	54230	2.58
**cl10444**	P-loop GTPases^**‡**^	198,624	115 (109)	3826672	464.67
**none**	AAA + ATPases^**‡**^	84,695	86(85)	1779227	173.73
**Started from unaligned sequences:**					
**none**	α,β- hydrolase fold	50,811	109(104)	752259	139.82
**none**	Helicases	86,287	117 (111)	1935380	342.10

^**‡**^ For these non-CDD curated alignments were used as input.

^**§**^ The time (in minutes) is for Steps 2 and 3 of the algorithm only.

Unaligned sequences were aligned using the multiple alignment procedures cited in Methods to generate an input alignment for the amcBPPS program.

## Conclusions

Currently the construction and annotation of CD hierarchies relies on the labor intensive process of manual curation. This has created a bottleneck hindering the CDD [53] from achieving the goal of comprehensive coverage of the protein domain universe. The incorporation of the amcBPPS program into the CDD curation pipeline can help automate this process while also providing a statistical measure of the quality of CD hierarchies. Likewise, the delete-half jackknife procedure applied here can provide qualitative estimates of the reliability of various features of a given hierarchy. And, because the amcBPPS program models protein domains based on those residue signatures that most distinguish each functionally divergent subgroup within a protein class from other subgroups, it can also accelerate the annotation of domain profiles. By linking these profiles to the Cn3D viewer [3,54] structural features associated with likely functionally critical residues can be identified within web-based BLAST searches. In previous studies (e.g., [55-59]) we have mapped key residues identified using the mcBPPS sampler to available crystal structures in this way, thereby obtaining insights into biological functions and mechanisms. Such information also facilitates structural evaluation of sequence alignments.

Of course, starting from the procedures described here, the CDD pipeline can be further automated and improved in various ways along similar lines. For example, we have demonstrated that our Bayesian alignment methods can be used to generate, for major protein classes (such as the AAA + ATPases, α,β-hydrolase fold enzymes and helicases in Table 2), large multiple alignments in the aligned block-based format required by the CDD. These, in turn, can serve as input alignments for generating protein domain hierarchies. Moreover, these alignment procedures could be refined to utilize information regarding pattern residue 3D structural interactions to identify and correct misaligned regions automatically (via iterative application of multiple alignment and BPPS procedures). Likewise, protein domain hierarchies generated by the amcBPPS program could be further optimized by implementing sampling operations to add or remove leaves and branches. More sophisticated taxonomic schemes could be devised for distinguishing conserved patterns due to functional constraints rather than to recent common descent. Taken together, these enhancements will accelerate the construction of an optimal, comprehensive set of hierarchically arranged CD profiles. This will free up curators to focus less on the tedious and labor intensive aspects of database construction and more on biological interpretation, a task that computational and statistical procedures cannot perform.

Having such a comprehensive set of well annotated, high quality CD profiles will summarize what is known about each type of domain. Through application of the MAPGAPS program, these CD hierarchies could be used to obtain up-to-date, very large and highly accurate multiple sequence alignments of an entire protein class for in-depth computational analyses. And by mapping various categories of pattern residues to corresponding structures, BLAST searches against these improved CD profiles can reveal those residues most likely responsible for the specific biochemical properties of a query protein. This can accelerate the pace of biological discovery by enabling researchers to obtain valuable clues regarding as-yet-unidentified protein biochemical and biophysical properties.

## Methods

Protein sequences were obtained from the NCBI nr and env_nr databases and from translated EST sequences within the NCBI est_others database (for which only open reading frames of at least 100 residues in length were retained). The phylum and kingdom to which each of these sequences belonged were determined using the NCBI taxonomy database dump. For those protein classes in Table 2 that lacked an existing curated alignment, sequences were identified through iterative PSI-BLAST [60] and PROBE [52] searches and then multiply aligned using a Bayesian MCMC multiple alignment method [51]. The MAPGAPS program [48] was used to obtain accurate multiple alignments containing vast numbers of sequences starting from a curated alignment. The mcBPPS sampling procedure is described in [42]. Routines to generate contrast alignments are described in [41].

### Evaluation procedures

The amcBPPS program was evaluated (see Table 1) as follows: First, an input multiple alignment for each domain was obtained using the alignments corresponding to the CDD hierarchies, as input to the MAPGAPS program [48]; this identified and aligned related sequences within the protein databases. (MAPGAPS aligns the sequences comparable to the accuracy of the curated alignments, which serve as templates.) The alignments obtained in this way were then used as input to the amcBPPS program to generate domain hierarchies. Each of these alignments was also used—along with the corresponding CDD seed alignments and FD-table (obtained from the tree, as shown in Figure 2)—as input to the mcBPPS sampler; this generates the same sort of hierarchy as is generated by the amcBPPS program. We then compared, for each domain, the consistency between the two output hierarchies—that is, we check whether the curated and automatically-generated FD-tables and seed alignments converged on more or less the same sequence sets (as illustrated in of Additional File 1: Figure S3). For the jackknife and simulation procedures the following domains (listed in Table 1) were used: cd00030, cd00138, cd00142, cd00159, cd00173, cd00229, cd00306, cd00368, cd00397, cd00768, cd00838, cd00900, cd01067, cd02156, cd02883, cd03128,cd03440, cd03873, cd05466, cd06587, cd06663, cd06846, cd08555, cd08772.

### Pseudocode for Step 1

The following pseudocode, which focuses on Step 1, corresponds to the main amcBPPS function, after which routines implementing Steps 2 and 3 are called. The output from Step 1 is used to create (in Step 2) a FD-table and a set of seed sequences for mcBPPS sampling (in Step 3). Note that this Step 1 pseudocode creates single category FD-tables, but it can be easily modified to create multiple category FD-tables.

**function** amcBPPS(*SeqAln*)//*creates a CD hierarchical alignment.*

**input:** a multiple alignment of protein sequences (*SeqAln*).

**output:** a hierarchy (tree) and corresponding contrast alignments (CHA).

//*assign each sequence to its own disjoint set* (*see Tarjan*[45])*.*

**for each** sequence *s* ∈ *SeqAln***do***s*.labeled := false; *s*.rank := ∞; Set(*s*) := {*s*}; **end for**

dheap *H*;//*Priority queue; for the data structure and algorithm see*[45]*.*

**for each** sequence pair < *s*_*1*_, *s*_*2*_ > **do**:

 **if** the sequences are from the same phylum **then**

  **if** sequences ≥ 95% identical then merge their disjoint sets **end if**

 **else if** sequences ≥ 40% identical **then**

  *key* := PercentIdentity(*s*_*1*_*, s*_*2*_);//*Using the pair-wise sequence identity as the key*…

  Insert(*key*, <*s*_*1*_, *s*_*2*_ >, *H*);// *store cross-phyla sequence pairs on priority queue*.

 **end if**

end for

//*Obtain an array of simple contrast alignments (CA) for distinct subgroups*.

*r* := 0; *g* := 0;

**while** < *s*_*1*_, *s*_*2*_ > := deleteMax(*H*) ≠ Ø **do**

 *r*++; *s*_*1*_.rank := min(*r, s*_*1*_.rank); *s*_*2*_.rank := min(*r, s*_*2*_.rank);

 **if** Â¬ *s*_*1*_.labeled ⋀ Â¬ *s*_*2*_.labeled ⋀ Set(*s*_*1*_) ≠ Set(*s*_*2*_) **then**

  Set(*s*_*1*_) := Set(*s*_*2*_) := Set(*s*_*1*_) ∩ Set(*s*_*2*_)*;*//*merge their sets*.

  **if** NumPhyla(Set(*s*_*1*_)) ≥ *N*_*min*_ then//*(by default, N*_*min*_ *= 4).*

   **for each***s* ∈ Set(*s*_*1*_) **do***s*.labeled := true; **end for**

   *g*++; *Seed*[*g*] := {};//*Seed set for group g.*

   **for each***p* ∈ {*p*: *p* = *s*.phylum ^ *s* ∈ Set(*s*_*1*_)*}***do**:

    //*Add to seed set the lowest ranked seq. from each phylum in merged set*.

    *Seed*[*g*] := *Seed*[*g*] ∩ {*s’*: *s’*.rank = min(*s’*.rank: *s’* ∈ Set(*s*_*1*_)⋀ *s’*.phylum = *p*)};

   **end for**

   *FD-tables*[*g*] := $\left[\begin{array}{c} +\\ +\\ - \end{array}\begin{array}{c} -\\ +\\ 0 \end{array}\right]$; //*column 2: subgroup g vs other proteins in class*.

   //*call mcBPPS sampler*[42]*to identify a contrast alignment for subgroup g*.

   *CHA*[*g*] := mcBPPS(*FD-tables*[*g*], *Seed*[g], *SeqAln*);

  **end if**

 **end if**

end while

*mc* := CreateFullHierarchy (*FD-tables*,*CHA*, g);*//Step 2: create mcBPPS input.*

**return** mcBPPS(*mc. FD-table*, *mc*.*Seed*, *SeqAln*)*//Step 3: optimize CD hierarchy.*

end function

Pseudocode for Step 2. Step 2 (i.e., the CreateFullHierarchy() routine) is subdivided into three sub-steps. For Step 2a, the MergeSimilarSets() function finds cliques of similar sequence sets by applying the Bron-Kerbosch algorithm [61] and then combines the sets within each clique:

**function** MergeSimilarSets(*SqSets*)

 **input:** sequence sets (*SqSets*) from Step 2.

 **output: **a reduced, non-redundant collection of sets and associated patterns.

 //*obtain an undirected graph of similar sequence sets*.

 Create a node for each input set

 **for each** pair of sets *I*, *J* within *SqSets***do**

  **if** the smaller set intersects with < 80% of the larger set **then** continue;

  Find pattern optimally discriminating sequences in sets I and J from other sequences;

  //*The optimum pattern is defined based on the mcBPPS statistical model*.

  **if** the two patterns intersect by < 33% or by < 5 pattern positions **then** continue;

  LLR_i,j_ := LLR with foreground = set I, background = , Â¬(set J ∩ set I) & set J pattern.

  LLR_j,i_ := LLR with foreground = set J, background =, Â¬(set J ∩ set I) & set I pattern.

  **if** LLR_i,j_ ≥ 80% of LLR_j,i_ ⋀ LLR_j,i_ ≥ 80% of LLR_i,j_**then** AddEdge(*I,J*) **end if**

 **end for**

 Find the cliques in the graph using the Bron-Kerbosch algorithm [61].

 **for each** clique **do**

  Create a consensus set of those sequences present in ≥ 50% of the clique sets.

  Compute pattern optimally discriminating consensus set from other sequences.

  Replace the sets belonging to the clique with the consensus set and pattern.

 **end for**

end function

By determining whether the sets substantially overlap, are roughly equal in size, and have similar discriminating patterns, the first two ‘if’ statement within MergeSimilarSets() merely prune the search by skipping over sets that are unlikely to correspond to the same protein subgroup. (Note that, if missed, sets corresponding to the same subgroup are likely to be detected in subsequent steps). To determine whether two different yet overlapping sets correspond to the same functionally-divergent subgroup, the procedure computes the BPPS log-likelihood using the pattern from one set with the partition defined by the other set and vice versa. If the patterns are more or less interchangeable between sets then an edge is added between the nodes corresponding to these sets. Next the Bron-Kerbosch algorithm is used to identify set cliques, each of which is then merged into a single (consensus) set. MergeSimilarSets() is applied iteratively to the modified sets from the previous iteration until it fails to identify and combine any additional similar sets.

Step 2b combines subgroup sets into larger supersets using the following FindSuperSets() function:

**function** FindSuperSets(*SqSets*)//*Obtain supersets from overlapping existing sets.*

 **input:** sequence sets (*SqSets*) from Step 2a.

 **for each** Set **do** Assign it to a unique disjoint set **end for***// (see Tarjan*[45]*).*

 **for each** pair of sets *I, J***do**//*find candidate supersets*

  **if** the intersection of the smaller set ≥ 66% of the larger set **then**

   Assign both sets to the same disjoint set;

  **endif**

 **end for**

 **for each** Disjoint set ‘dset’ containing at least 2 subsets **do**

  Superset := the union of the subsets;

  Superpattern := the pattern optimally discriminating the Superset from Â¬ Superset;

  **if** Any subsets in dset fail to contribute their *‘fair share’* to the superset LLR **then**

   Remove these subsets from dset and repeat from the start of this ‘for’ loop

  **else** Save the superset and superpattern **endif**

 **end for**

 **return:** The saved supersets and superpatterns.

end function

FindSuperSets() first identifies collections of (possibly minimally) overlapping sequence sets as possible candidates for merging into supersets. Next, it combines into a superset those sets that contribute their ‘fair share’ to the optimum LLR for the proposed superset—where the ‘fair share’ is defined as contributing at least 80% of the estimated average contribution of each sequence to the LLR times the number of sequences in the subset. (Based on the statistical formulation [40,42], each sequence will contribute equally, on average, to the log-likelihood. For such calculations, however, the sequences are down-weighted for redundancy, as previously described [40]).

Next the function CreateSuperSets() is called to create additional supersets from the current sets that fail to overlap or that overlap only moderately. As long as new supersets are created, this function is called repeatedly (this merges subsets into supersets that might otherwise have been overlooked).

**function** CreateSuperSets(*SqSets*)//Create supersets by combining (possibly distinct) sets.

 **input**: sequence sets (*SqSets*).

 **output**: new supersets.

 **for each** set I **do**

  SuperSet := set I; SuperPattern := Ø;

  **for each** set J that at least slightly overlaps with set I **do**

   Set X := SuperSet ∩ set J;

   Pattern X := the pattern optimally discriminating set X from Â¬ X;

   **if** both SuperSet & set J contribute their *‘fair share’* a significant LLR **then**

   Superset := Set X; SuperPattern := pattern;

   **endif**

  **end for**

  **if** set I ⊂ SuperSet **then** save the current Superset **endif**

 **end for**

end function

Step 2c uses the sets obtained in the previous steps to construct a tree hierarchy, from which a FD-table is then obtained—, along with corresponding seed alignments and initial partitions—as follows:

**function** CreateTree(*SqSets*)//*obtain an optimized tree*.

 **input:** sequence sets (*SqSets*) and corresponding patterns from steps 1–2 above.

 **output:** a FD-table + corresponding starting subgroup sets, patterns, and seed alignments

 wdiGrph := RtnDiGraph(*SqSets*);//*returns a weighted directed graph of set relationships*

 Tree := ShortestPathTree(wdiGrph);//*as defined by Tarjan*[45]

 Tree := RefineTree(Tree);//*eliminates insignificant nodes and overlap between sets.*

 FD-Table := TreeToFDtable(Tree);

 sma := CreateSeedAlignments(Tree); // *characteristic, cross-phyla seqs for each set*.

end function

where the RtnDiGraph() functions is defined as:

**function** RtnDiGraph (*SqSets*)

 **input:** sequence sets (*SqSets*) from Steps 2a and 2b.

 **output:** a weighted directed acyclic graph representing the set relationships.

 Create a weighted directed graph where each set is a node

 **for each** pair of sets *I, J***do**//*find pairs of sets where set I ⊂ set J*.

  *//simple heuristics for speed*.

  **if** setI is smaller than setJ **then** continue

  **else if** setI ∩ setJ < 50% of setI **then** continue

  **else if** setJ < 33% larger than setI ∩ setJ **then** continue **endif**

  *//identify those pairs where set I is a (typically fuzzy) subset of set J*

  Compute the optimum pattern and LLR for set I versus set J – set I;

  **if** LLR is not significant then continue;//*significance defined as for FindSuperSets ()*

  **if** Set I fails to contribute its *‘fair share’* to the superset LRR **then** continue;

  Add an arc pointing from node J to node I & weighted by –LLR;

 **end for**

 **for each** set that lacks a Superset **do**

  Compute the optimum pattern and LLR for the set versus the complementary set;

  Add an arc pointing from the root to the corresponding node & weighted by –LLR

 **end for**

end function

Note that the RtnDiGraph () function returns a directed acyclic graph (DAG), for which the ShortestPathTree() algorithm [45] finds a minimum spanning tree emanating from the root node. Because the distances assigned to the arcs in the graph correspond to the negatives of the LLRs, this tree maximizes the total LLR as defined for the corresponding FD-table (see [42]). Incidentally, in this sense, this approach is akin to using the data to infer the DAG and parameters corresponding to a Bayesian network [62] and then determining the most likely paths through the DAG from a predefined root node. This approach avoids the computational expense of using MCMC sampling to optimally define both the FD-table and the corresponding pattern-partition pairs concurrently by using an heuristic approach that is substantially faster yet still based on statistical criteria.

The sequence sets corresponding to the tree returned by the ShortestPathTree() algorithm are still fuzzily defined and thus typically contain sequences that belong to one or more distinct protein subgroups and thus that are not proper subsets of their respective supersets. The following RefineTree() function eliminates inappropriate overlap between sets while also eliminating nodes from the tree that, as a result of the refinement process, are no longer statistically significant:

**function** RefineTree (*Tree*)//*return a refined tree representing subgroup relationships*.

 **input:** a tree where each node corresponds to a sequence set

 **output:** refined tree

 **do**

  **do**//*eliminate insignificant nodes from the tree…*

   Find the arc with the lowest weight (i.e., with the lowest subset-to-superset LLR);

   **if** this LLR is not significant **then**

    Remove the arc and the child (subset) node from the tree;

    Connect the children of the removed node to the parent of that node;

    Merge the set corresponding to the removed node into the parent set;

   **end if**

  **while** an arc has been removed;

  **do** //*eliminate overlap between the sequence sets…*

   Label the leaf nodes as ‘candidates’ and leave other nodes unlabeled.

   **for each** pair of nodes **do**

    **if**both nodes are labeled as ‘fixed’ **then** continue;

    **else if** one node is the root **then** continue;

    **else if** one node is ‘fixed’ and the other is a ‘candidate’ **then**

     remove all overlapping sequences from ‘candidate’ node;

    **else if** both nodes are candidates **then**

     **for each** sequence *S* present in both node sets **do**

      remove *S* from the set with the poorer optimal pattern match;

     **end for**

    **end if**

   **end for**

   Label all current ‘candidate’ nodes as ‘fixed’;

   Label as ‘candidates’ all nodes whose subtree consists entirely of labeled nodes;

  **while** some nodes were newly labeled as candidates;

  Define the root node set as containing all sequences absent from the other node sets;

  Merge each leaf node with only a few sequences into its parent node;

  Merge nodes with a single child into their parent nodes; //*this step is optional*

  Relocate nodes that, due to previous step, are no longer properly placed in the tree.

 **while** the tree has been changed in any way;

end function

The tree returned by the RefineTree() function is output as a Newick-format character string (a formal language specification for trees), which is then parsed and translated into a FD-table within the CreateTree() routine. This routine also creates a seed alignment for each row in the FD-table using a few of the most characteristic sequences in each set. These, along with the corresponding patterns (one for each column), are then used as input to the mcBPPS procedure (Step 3).

## Abbreviations

*CD*: conserved domain; *CDD*: Conserved Domain Database; *DAG*: directed acyclic graph; *FD*: functional divergence; *LLR*: log-likelihood ratio; *mcBPPS*: multiple category Bayesian Partitioning with Pattern Selection; *amcBPPS*: automated mcBPPS; *MCMC*: Markov chain Monte Carlo.

## Competing interests

The authors declare that they have no competing interests.

## Authors’ contributions

AFN designed and implemented the algorithm, performed the jackknife analyses and simulations, generated the multiple sequence alignments used as input to the amcBPPS program, ran the programs and wrote the initial draft of the manuscript. CL and AMB converted CDD alignments and hierarchies into appropriate formats for analysis and provided additional CDD information as required for this study. All authors evaluated the output files and read, revised and approved the manuscript.

## Supplementary Material

Additional File 1 Additional figures referred to in the main article as Figures S1–S6. Click here for file
