# Supplementary material for: Automated hierarchical classification of protein domain subfamilies based on functionally-divergent residue signatures
Source: BMC Bioinformatics. 2012 Jun 22;13:144. doi: 10.1186/1471-2105-13-144 (PMC3599474; doi:10.1186/1471-2105-13-144)

Automatic generation of protein domain hierarchies based on functionally-divergent residue signatures

Additional File 1

Andrew F. Neuwald, Christopher J. Lanczycki and Aron Marchler-Bauer

Additional figures referred to in the main article as Figures S1–S6.

Figure S1. **Output contrast hierarchical alignments**.

Figure S2. **Recursive analysis of the Ras-like subgroup** .

Figure S3. **Output of our program for comparing two hierarchies**.

Figure S4. **An example output file from our jackknife procedure**.

Figure S5. **An example output file from our simulation procedure**.

Figure S6. **A CDD "sequence tree" computed from the amcBPPS hierarchy for RRM domains**.

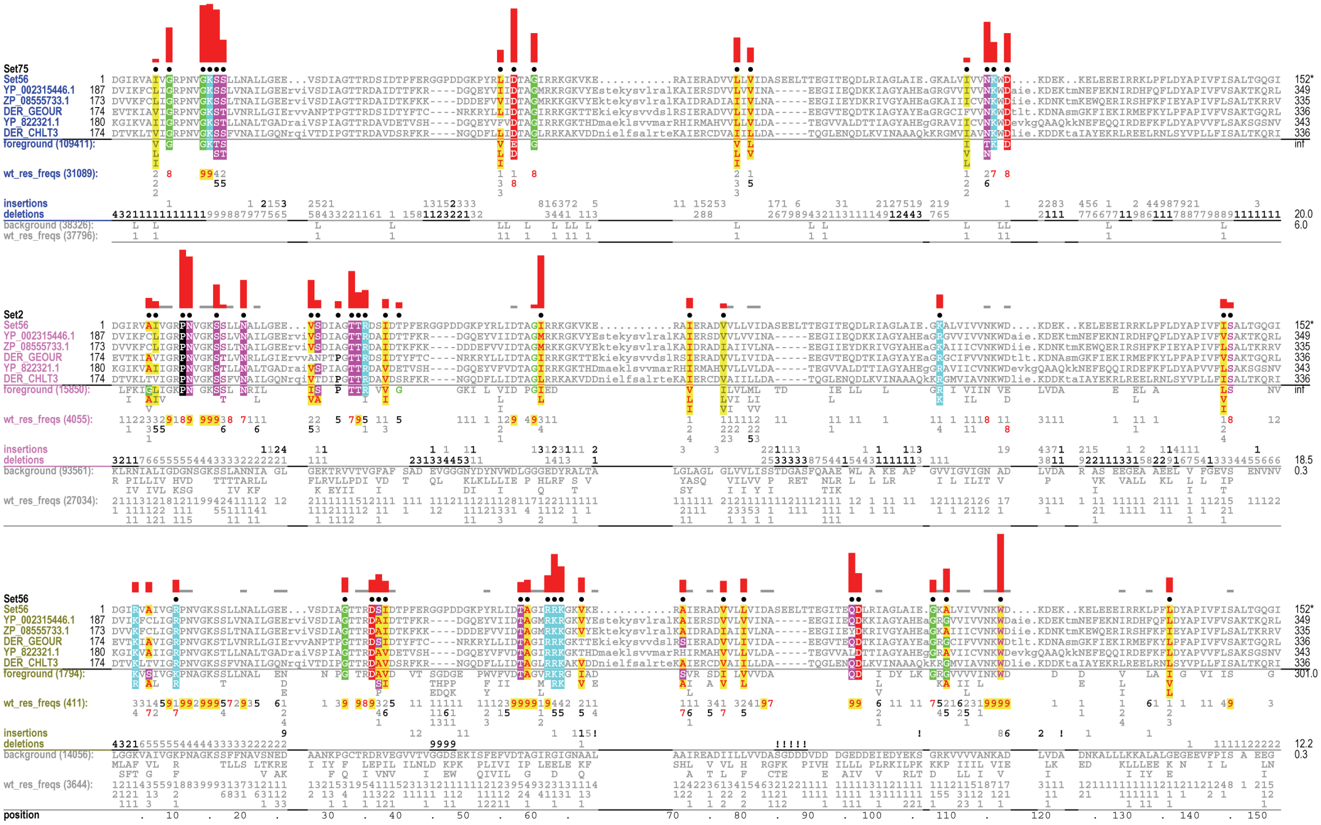

Figure S1. **Output contrast hierarchical alignments**

The alignments shown correspond to row 36 in Figure 4 of the main article and highlight a consensus and five representative sequences in three ways to reveal patterns most distinctive of three functionally-divergent subgroups to which these belong: (**top**) patterns distinguishing all P-loop GTPases from other proteins; (**middle**) patterns distinguishing subset #2 from other -loop GTPases; and (**bottom**) patterns distinguishing subset #56 from other subset #2 sequences. Characteristic foreground residues at each position are shown below each alignment and, directly below these, corresponding frequencies are given in integer tenths. A ‘7’, for example, indicates that the corresponding residue occurs in 70-80% of the sequences. Below these, corresponding background residues and frequencies are shown in gray. The heights of red bars above highlighted columns quantify the selective pressure imposed on pattern positions. Black dots indicate pattern positions selected by the sampler.

Figure S2. **Recursive analysis of the Ras-like subgroup** .
This corresponds to rows 18-26 in Figure 4 of the main article.

================ HyperPartition: ================

Category

Set:	1	2	3	4	5	6	7	8	9	10	11	12	13	14	15	16		
1:	+	-	-	-	o	o	o	o	o	o	o	o	-	-	-	-	Set29 (4410)	
2:	+	+	-	-	o	o	o	o	o	o	o	o	-	-	-	-	Set27 (59)	ypt
3:	+	-	+	-	o	o	o	o	o	o	o	o	-	-	-	-	Set25 (207)	DiRas2
4:	+	-	-	+	-	-	-	-	o	o	-	-	-	-	-	-	Set28 (3725)	Ras/Ran/Rab
5:	+	-	-	+	+	-	-	-	o	o	-	-	-	-	-	-	Set18 (1431)	Rab5-like
6:	+	-	-	+	-	+	-	-	o	o	-	-	-	-	-	-	Set24 (72)	Rab34
7:	+	-	-	+	-	-	+	-	o	o	-	-	-	-	-	-	Set22 (68)	Rab33
8:	+	-	-	+	-	-	-	+	-	-	-	-	-	-	-	-	Set2 (4003)	Rab-family
9:	+	-	-	+	-	-	-	+	+	-	-	-	-	-	-	-	Set17 (802)	Rab14
10:	+	-	-	+	-	-	-	+	-	+	-	-	-	-	-	-	Set15 (2207)	Rab-famB?
11:	+	-	-	+	-	-	-	-	o	o	+	-	-	-	-	-	Set14 (1166)	RabG
12:	+	-	-	+	-	-	-	-	o	o	-	+	-	-	-	-	Set12 (542)	RabH
13:	+	-	-	-	o	o	o	o	o	o	o	o	+	-	-	-	Set7 (2723)	Rho-family
14:	+	-	-	-	o	o	o	o	o	o	o	o	-	+	-	-	Set6 (77)	RabDnaJ
15:	+	-	-	-	o	o	o	o	o	o	o	o	-	-	+	-	Set5 (83)	Rab28
16:	+	-	-	-	o	o	o	o	o	o	o	o	-	-	-	+	Set4 (68)	IFT27
17:	-	o	o	o	o	o	o	o	o	o	o	o	o	o	o	o	Rejected (9)	

==

Figure S3. **Output of our program for comparing two hierarchies**.

This reveals the degree to which the curated and automatic hierarchies for the PI3Kc_like domain (cd00142) overlap. Shown in parentheses are both the numbers of sequences at each level of the hierarchy and the number at that level assigned to various level of the other hierarchy. The common subgroups are color coded between the two hierarchies (i.e., FD-tables) for clarity.

```
Automated vs curated sets:
  +-ooooo-oo--ooo-    1.Set21? (86)-> cd00896(15) cd05172(4) [60 parent; 1 reject] (3 other internal) + 3.
  ++-oo---oo--ooo-    2.Set1? (114)-> cd05169(54) cd05170(13) cd00892(6) [40 parent] + 1.
  +++-----oo--ooo-    3.Set11? (1)-> cd05171(1).
  ++++----oo--ooo-    4.Set17! (194)-> cd00892(189) [5 parent].
  +++-+---oo--ooo-    5.Set16! (187)-> cd05171(185) [2 parent].
  ++-oo+--oo--ooo-    6.Set12! (244)-> cd05169(244).
  ++-oo-+-oo--ooo-    7.Set9! (54)-> cd05170(54).
  +-ooooo+----ooo-    8.Set3? (65)-> cd05167(24) cd05168(23) [18 parent].
  +-ooooo++---ooo-    9.Set10! (252)-> cd05167(252).
  +-ooooo+-+--ooo-   10.Set8! (301)-> cd05168(296) [5 parent].
  +-ooooo-oo+-ooo-   11.Set7! (180)-> cd05163(180).
  +-ooooo-oo-+----   12.Set2? (33)-> cd00894(2) cd05175(2) (29 other internal).
  +-ooooo-oo-++---   13.Set4! (103)-> cd05177(21) cd00895(20) cd05176(19) [43 parent].
  +-ooooo-oo-+-+--   14.Set18! (202)-> cd00894(22) cd05174(17) cd05173(16) cd05175(15) [132 parent].
  +-ooooo-oo-+--+-   15.Set6! (255)-> cd00896(255).
  +-ooooo-oo--ooo+   16.Set5! (68)-> cd05172(65) [3 parent].
  -ooooooooooooooo   17.Reject (70)-> cd05163(35) [2 parent; 31 reject] (2 other internal).

 Inverse comparison:
  +-oooooooooo-oo--ooooo    1.cd00142? (59)-> Set5(3) Set16(2) [51 parent; 2 reject] + 1.
  ++--oooo-ooo-oo--ooooo    2.cd00891? (56)-> Set18(34) [22 parent].
  +++-oooo-ooo-oo--ooooo    3.cd00896! (270)-> Set6(255) [15 parent].
  ++-+-----ooo-oo--ooooo    4.cd05165? (104)-> Set18(98) [6 parent].
  ++-++----ooo-oo--ooooo    5.cd00894! (24)-> Set18(22) [2 parent].
  ++-+-+---ooo-oo--ooooo    6.cd05173! (16)-> Set18(16).
  ++-+--+--ooo-oo--ooooo    7.cd05174! (17)-> Set18(17).
  ++-+---+-ooo-oo--ooooo    8.cd05175! (17)-> Set18(15) [2 parent].
  ++--oooo+----oo--ooooo    9.cd05166? (47)-> Set4(43) [4 parent].
  ++--oooo++---oo--ooooo   10.cd00895! (20)-> Set4(20).
  ++--oooo+-+--oo--ooooo   11.cd05176! (19)-> Set4(19).
  ++--oooo+--+-oo--ooooo   12.cd05177! (21)-> Set4(21).
  +-oooooooooo+----ooooo   13.cd00893? (25)-> Set8(4) [21 parent].
  +-oooooooooo++---ooooo   14.cd05167! (276)-> Set10(252) [24 parent].
  +-oooooooooo+-+--ooooo   15.cd05168! (319)-> Set8(296) [23 parent].
  +-oooooooooo-oo+-ooooo   16.cd05163! (215)-> Set7(180) [35 reject].
  +-oooooooooo-oo-+-----   17.cd05164? (53)-> Set17(5) [46 parent; 2 reject].
  +-oooooooooo-oo-++----   18.cd00892! (196)-> Set17(189) [7 parent].
  +-oooooooooo-oo-+-+---   19.cd05169! (298)-> Set12(244) [54 parent].
  +-oooooooooo-oo-+--+--   20.cd05170! (68)-> Set9(54) [14 parent].
  +-oooooooooo-oo-+---+-   21.cd05171! (188)-> Set16(185) [3 parent].
  +-oooooooooo-oo-+----+   22.cd05172! (69)-> Set5(65) [4 parent].
  -ooooooooooooooooooooo   23.Reject (32)-> [31 reject] (1 other internal).
```

Figure S4. **An example output file from our jackknife procedure**.

The output shown is from an analysis of PH-domains (cd00900), which are particularly challenging given the large number of nodes and sequences in the hierarchy. A delete-half jackknife resampling procedure was used where half of the 22,593 sequences in the original input alignment were randomly discarded leaving 11,296 in the alignment which is then used as input to the amcBPPS program to obtain a jackknife hierarchy. Shown are two such hierarchies, labeled ‘A’ and ‘B’, consisting of 91 and 76 nodes, respectively. Each line corresponds to a node sequence set (labeled as ‘Set<id>’) with the number of sequences in that set (and that are also present in the other hierarchy) in parentheses; after the colon on each line are listed those node sets in the other hierarchy sharing one or more of the sequences in that set (with the number of sequences likewise in parentheses). These two hierarchies share 5,678 sequences in common. This comparison reveals that, among 59 leaf node sets in tree A that share at least one sequence in common with a leaf node set in tree B, 42 (or 71%) share all of their sequences in common with a tree B node (i.e., among those sequences present in both trees) and 48 (81%) share >90% of their sequences with a tree B node. In cases where identical sequence sets are not found, the ‘missing’ sequences were typically assigned to a parent or root node further up the tree (or were rejected) rather than to an unrelated leaf node in the other tree.

Hierarchy A:

```
+------------------------oooo-------------ooooooo-ooo--------ooo-----oo-oooooooo---ooooo---   1.Set173? (708): Set134(70) Set148(12) Set71(11) Set119(7) [519 parent; 83 reject] + 6.
++-----------------------oooo-------------ooooooo-ooo--------ooo-----oo-oooooooo---ooooo---   2.Set133! (19): Set65(15) [4 parent].
+-+----------------------oooo-------------ooooooo-ooo--------ooo-----oo-oooooooo---ooooo---   3.Set71! (39): Set161(39).
+--+---------------------oooo-------------ooooooo-ooo--------ooo-----oo-oooooooo---ooooo---   4.Set161! (20): Set153(20).
+---+--------------------oooo-------------ooooooo-ooo--------ooo-----oo-oooooooo---ooooo---   5.Set103! (21): Set158(20) [1 parent].
+----+-------------------oooo-------------ooooooo-ooo--------ooo-----oo-oooooooo---ooooo---   6.Set86! (75): New! [75 reject].
+-----+------------------oooo-------------ooooooo-ooo--------ooo-----oo-oooooooo---ooooo---   7.Set65! (20): Set63(20).
+------+-----------------oooo-------------ooooooo-ooo--------ooo-----oo-oooooooo---ooooo---   8.Set48! (457): Set35(454) [3 reject].
+-------+----------------oooo-------------ooooooo-ooo--------ooo-----oo-oooooooo---ooooo---   9.Set163! (13): Set134(9) [4 parent].
+--------+---------------oooo-------------ooooooo-ooo--------ooo-----oo-oooooooo---ooooo---   10.Set147! (23): Set114(23).
+---------+--------------oooo-------------ooooooo-ooo--------ooo-----oo-oooooooo---ooooo---   11.Set144! (44): Set134(31) Set148(7) [5 parent; 1 reject].
+----------+-------------oooo-------------ooooooo-ooo--------ooo-----oo-oooooooo---ooooo---   12.Set32! (17): New! [1 reject] (16 other internal).
+-----------+------------oooo-------------ooooooo-ooo--------ooo-----oo-oooooooo---ooooo---   13.Set135! (32): New! [32 reject].
+------------+-----------oooo-------------ooooooo-ooo--------ooo-----oo-oooooooo---ooooo---   14.Set24! (33): Set31(22) [11 reject].
+-------------+----------oooo-------------ooooooo-ooo--------ooo-----oo-oooooooo---ooooo---   15.Set122! (18): Set83(18).
+--------------+---------oooo-------------ooooooo-ooo--------ooo-----oo-oooooooo---ooooo---   16.Set120! (27): Set145(27).
+---------------+--------oooo-------------ooooooo-ooo--------ooo-----oo-oooooooo---ooooo---   17.Set119! (24): Set40(23) [1 parent].
+----------------+-------oooo-------------ooooooo-ooo--------ooo-----oo-oooooooo---ooooo---   18.Set115! (15): Set66(15).
+-----------------+------oooo-------------ooooooo-ooo--------ooo-----oo-oooooooo---ooooo---   19.Set111! (14): Set54(14).
+------------------+-----oooo-------------ooooooo-ooo--------ooo-----oo-oooooooo---ooooo---   20.Set110! (26): Set90(26).
+-------------------+----oooo-------------ooooooo-ooo--------ooo-----oo-oooooooo---ooooo---   21.Set109! (20): New! (20 other internal).
+--------------------+---oooo-------------ooooooo-ooo--------ooo-----oo-oooooooo---ooooo---   22.Set104! (13): New! [1 reject] (12 other internal).
+---------------------+--oooo-------------ooooooo-ooo--------ooo-----oo-oooooooo---ooooo---   23.Set102! (17): New! (17 other internal).
+----------------------+-oooo-------------ooooooo-ooo--------ooo-----oo-oooooooo---ooooo---   24.Set100! (21): Set84(21).
+-----------------------+--o--------------ooooooo-ooo--------ooo-----oo-oooooooo---ooooo---   25.Set9? (78): Set150(1) [15 reject] (61 other internal) + 1.
+-----------------------++-o--------------ooooooo-ooo--------ooo-----oo-oooooooo---ooooo---   26.Set36! (53): Set69(26) Set72(23) [4 parent].
+-----------------------+-+---------------ooooooo-ooo--------ooo-----oo-oooooooo---ooooo---   27.Set105? (13): (13 other internal).
+-----------------------+-++--------------ooooooo-ooo--------ooo-----oo-oooooooo---ooooo---   28.Set96! (15): Set140(15).
+-----------------------+--o+-------------ooooooo-ooo--------ooo-----oo-oooooooo---ooooo---   29.Set140! (13): Set150(13).
+------------------------oooo+------------ooooooo-ooo--------ooo-----oo-oooooooo---ooooo---   30.Set93! (17): New! (17 other internal).
+------------------------oooo-+-----------ooooooo-ooo--------ooo-----oo-oooooooo---ooooo---   31.Set22! (17): Set56(17).
+------------------------oooo--+----------ooooooo-ooo--------ooo-----oo-oooooooo---ooooo---   32.Set91! (13): New! (13 other internal).
+------------------------oooo---+---------ooooooo-ooo--------ooo-----oo-oooooooo---ooooo---   33.Set90! (17): Set93(17).
+------------------------oooo----+--------ooooooo-ooo--------ooo-----oo-oooooooo---ooooo---   34.Set88! (11): New! [4 reject] (7 other internal).
+------------------------oooo-----+-------ooooooo-ooo--------ooo-----oo-oooooooo---ooooo---   35.Set87! (16): Set134(13) [3 parent].
+------------------------oooo------+------ooooooo-ooo--------ooo-----oo-oooooooo---ooooo---   36.Set84! (11): New! (11 other internal).
+------------------------oooo-------+-----ooooooo-ooo--------ooo-----oo-oooooooo---ooooo---   37.Set83! (29): Set68(29).
+------------------------oooo--------+----ooooooo-ooo--------ooo-----oo-oooooooo---ooooo---   38.Set82! (21): Set75(21).
+------------------------oooo---------+---ooooooo-ooo--------ooo-----oo-oooooooo---ooooo---   39.Set77! (30): New! (30 other internal).
+------------------------oooo----------+--ooooooo-ooo--------ooo-----oo-oooooooo---ooooo---   40.Set76! (11): New! (11 other internal).
+------------------------oooo-----------+-ooooooo-ooo--------ooo-----oo-oooooooo---ooooo---   41.Set73! (32): Set73(25) [4 parent; 3 reject].
+------------------------oooo------------+-oo-ooo-ooo--------ooo-----oo-oooooooo---ooooo---   42.Set34? (2): [2 reject].
+------------------------oooo------------++---ooo-ooo--------ooo-----oo-oooooooo---ooooo---   43.Set13? (25): Set167(2) [23 reject].
+------------------------oooo------------+++--ooo-ooo--------ooo-----oo-oooooooo---ooooo---   44.Set132! (17): New! [17 reject].
+------------------------oooo------------++-+-ooo-ooo--------ooo-----oo-oooooooo---ooooo---   45.Set81! (17): Set167(17).
+------------------------oooo------------+-oo+-o--ooo--------ooo-----oo-oooooooo---ooooo---   46.Set37? (15): [15 reject].
+------------------------oooo------------+-oo++---ooo--------ooo-----oo-oooooooo---ooooo---   47.Set57? (16): [16 reject].
+------------------------oooo------------+-oo+++--ooo--------ooo-----oo-oooooooo---ooooo---   48.Set124! (22): Set98(22).
+------------------------oooo------------+-oo+-o+-ooo--------ooo-----oo-oooooooo---ooooo---   49.Set170! (28): Set166(28).
+------------------------oooo-------------ooooooo+-----------ooo-----oo-oooooooo---ooooo---   50.Set18? (925): Set134(43) Set15(17) Set20(17) Set97(17) [792 parent; 20 reject] (2 other internal) + 17.
+------------------------oooo-------------ooooooo++----------ooo-----oo-oooooooo---ooooo---   51.Set164! (16): Set128(16).
+------------------------oooo-------------ooooooo+-+---------ooo-----oo-oooooooo---ooooo---   52.Set97! (38): New! [2 reject] (36 other internal).
+------------------------oooo-------------ooooooo+--+--------ooo-----oo-oooooooo---ooooo---   53.Set70! (14): Set139(14).
+------------------------oooo-------------ooooooo-ooo+-------ooo-----oo-oooooooo---ooooo---   54.Set21! (23): New! [23 reject].
+------------------------oooo-------------ooooooo-ooo-+------ooo-----oo-oooooooo---ooooo---   55.Set68! (50): Set53(42) [5 parent; 3 reject].
+------------------------oooo-------------ooooooo-ooo--+-----ooo-----oo-oooooooo---ooooo---   56.Set67! (25): Set51(25).
+------------------------oooo-------------ooooooo-ooo---+----ooo-----oo-oooooooo---ooooo---   57.Set66! (27): Set50(27).
+------------------------oooo-------------ooooooo-ooo----+---ooo-----oo-oooooooo---ooooo---   58.Set64! (23): Set47(23).
+------------------------oooo-------------ooooooo-ooo-----+--ooo-----oo-oooooooo---ooooo---   59.Set63! (42): Set62(41) [1 parent].
+------------------------oooo-------------ooooooo-ooo------+-ooo-----oo-oooooooo---ooooo---   60.Set62! (51): Set48(50) [1 reject].
+------------------------oooo-------------ooooooo-ooo-------+--------oo-oooooooo---ooooo---   61.Set41? (0).
+------------------------oooo-------------ooooooo-ooo-------++-------oo-oooooooo---ooooo---   62.Set134! (26): Set20(26).
+------------------------oooo-------------ooooooo-ooo-------+-+------oo-oooooooo---ooooo---   63.Set61! (19): Set49(19).
+------------------------oooo-------------ooooooo-ooo-------+--+-----oo-oooooooo---ooooo---   64.Set171! (13): Set20(13).
+------------------------oooo-------------ooooooo-ooo--------ooo+----oo-oooooooo---ooooo---   65.Set60! (33): Set44(33).
+------------------------oooo-------------ooooooo-ooo--------ooo-+---oo-oooooooo---ooooo---   66.Set59! (19): Set61(19).
+------------------------oooo-------------ooooooo-ooo--------ooo--+--oo-oooooooo---ooooo---   67.Set56! (32): Set41(32).
+------------------------oooo-------------ooooooo-ooo--------ooo---+-oo-oooooooo---ooooo---   68.Set54! (21): Set81(21).
+------------------------oooo-------------ooooooo-ooo--------ooo----+-o-oooooooo---ooooo---   69.Set28? (580): Set114(1) [21 reject] (556 other internal) + 2.
+------------------------oooo-------------ooooooo-ooo--------ooo----++--oooooooo---ooooo---   70.Set74? (106): Set102(106).
+------------------------oooo-------------ooooooo-ooo--------ooo----+++-oooooooo---ooooo---   71.Set53! (0).
+------------------------oooo-------------ooooooo-ooo--------ooo-----oo+----oo-----ooooo---   72.Set26? (206): Set137(28) Set45(3) Set86(3) [107 parent; 62 reject] (2 other internal) + 1.
+------------------------oooo-------------ooooooo-ooo--------ooo-----oo++---oo-----ooooo---   73.Set58! (39): Set57(39).
+------------------------oooo-------------ooooooo-ooo--------ooo-----oo+-+--oo-----ooooo---   74.Set114! (21): Set108(21).
+------------------------oooo-------------ooooooo-ooo--------ooo-----oo+--+-oo-----ooooo---   75.Set160! (26): Set77(26).
+------------------------oooo-------------ooooooo-ooo--------ooo-----oo+---+-------ooooo---   76.Set10? (76): Set45(32) [44 parent].
+------------------------oooo-------------ooooooo-ooo--------ooo-----oo+---++------ooooo---   77.Set51! (28): New! (28 other internal).
+------------------------oooo-------------ooooooo-ooo--------ooo-----oo+---+-+-----ooooo---   78.Set127! (21): Set45(21).
+------------------------oooo-------------ooooooo-ooo--------ooo-----oo+----oo+----ooooo---   79.Set95! (19): Set86(19).
+------------------------oooo-------------ooooooo-ooo--------ooo-----oo+----oo-+---ooooo---   80.Set121! (21): Set64(21).
+------------------------oooo-------------ooooooo-ooo--------ooo-----oo-oooooooo+--ooooo---   81.Set49! (31): Set39(31).
+------------------------oooo-------------ooooooo-ooo--------ooo-----oo-oooooooo-+-ooooo---   82.Set47! (140): Set36(140).
+------------------------oooo-------------ooooooo-ooo--------ooo-----oo-oooooooo--+-oo-----   83.Set7? (57): Set92(13) [42 parent; 2 reject].
+------------------------oooo-------------ooooooo-ooo--------ooo-----oo-oooooooo--++-------   84.Set15? (0).
+------------------------oooo-------------ooooooo-ooo--------ooo-----oo-oooooooo--+++------   85.Set149! (19): Set92(19).
+------------------------oooo-------------ooooooo-ooo--------ooo-----oo-oooooooo--++-+-----   86.Set89! (29): Set21(13) [16 parent].
+------------------------oooo-------------ooooooo-ooo--------ooo-----oo-oooooooo--+-oo+----   87.Set45! (30): Set38(30).
+------------------------oooo-------------ooooooo-ooo--------ooo-----oo-oooooooo--+-oo-+---   88.Set98! (21): Set92(17) [4 parent].
+------------------------oooo-------------ooooooo-ooo--------ooo-----oo-oooooooo---ooooo+--   89.Set31! (49): Set41(45) [4 parent].
+------------------------oooo-------------ooooooo-ooo--------ooo-----oo-oooooooo---ooooo-+-   90.Set23! (18): Set105(18).
+------------------------oooo-------------ooooooo-ooo--------ooo-----oo-oooooooo---ooooo--+   91.Set154! (20): Set70(17) [3 reject].
-oooooooooooooooooooooooooooooooooooooooooooooooooooooooooooooooooooooooooooooooooooooooooo   92.Reject (469): Set91(18) Set76(16) Set134(8) Set86(7) [95 parent; 301 reject] (10 other internal) + 14.
```

Figure S4 (cont.).

Hierarchy B:

```
+----------------------ooo---oo-----ooo----ooo------ooooooooo-----ooo------   1.Set170? (2174): Set97(36) Set77(30) Set109(20) Set102(17) [1290 parent; 95 reject] (584 other internal) + 102.
++---------------------ooo---oo-----ooo----ooo------ooooooooo-----ooo------   2.Set158! (20): Set103(20).
+-+--------------------ooo---oo-----ooo----ooo------ooooooooo-----ooo------   3.Set153! (20): Set161(20).
+--+-------------------ooo---oo-----ooo----ooo------ooooooooo-----ooo------   4.Set150! (14): Set140(13) [1 parent].
+---+------------------ooo---oo-----ooo----ooo------ooooooooo-----ooo------   5.Set145! (27): Set120(27).
+----+-----------------ooo---oo-----ooo----ooo------ooooooooo-----ooo------   6.Set128! (16): Set164(16).
+-----+----------------ooo---oo-----ooo----ooo------ooooooooo-----ooo------   7.Set90! (26): Set110(26).
+------+---------------ooo---oo-----ooo----ooo------ooooooooo-----ooo------   8.Set86! (29): Set95(19) [3 parent; 7 reject].
+-------+--------------ooo---oo-----ooo----ooo------ooooooooo-----ooo------   9.Set75! (21): Set82(21).
+--------+-------------ooo---oo-----ooo----ooo------ooooooooo-----ooo------   10.Set148! (26): Set144(7) [12 parent; 1 reject] (6 other internal).
+---------+------------ooo---oo-----ooo----ooo------ooooooooo-----ooo------   11.Set134! (174): Set144(31) Set87(13) Set163(9) [70 parent; 8 reject] (43 other internal).
+----------+-----------ooo---oo-----ooo----ooo------ooooooooo-----ooo------   12.Set114! (24): Set147(23) (1 other internal).
+-----------+----------ooo---oo-----ooo----ooo------ooooooooo-----ooo------   13.Set105! (23): Set23(18) [2 parent; 1 reject] (2 other internal).
+------------+---------ooo---oo-----ooo----ooo------ooooooooo-----ooo------   14.Set97! (18): New! (18 other internal).
+-------------+--------ooo---oo-----ooo----ooo------ooooooooo-----ooo------   15.Set93! (17): Set90(17).
+--------------+-------ooo---oo-----ooo----ooo------ooooooooo-----ooo------   16.Set91! (19): New! [18 reject] (1 other internal).
+---------------+------ooo---oo-----ooo----ooo------ooooooooo-----ooo------   17.Set84! (21): Set100(21).
+----------------+-----ooo---oo-----ooo----ooo------ooooooooo-----ooo------   18.Set83! (19): Set122(18) [1 parent].
+-----------------+----ooo---oo-----ooo----ooo------ooooooooo-----ooo------   19.Set81! (24): Set54(21) [1 reject] (2 other internal).
+------------------+---ooo---oo-----ooo----ooo------ooooooooo-----ooo------   20.Set76! (16): New! [16 reject].
+-------------------+--ooo---oo-----ooo----ooo------ooooooooo-----ooo------   21.Set73! (25): Set73(25).
+--------------------+-ooo---oo-----ooo----ooo------ooooooooo-----ooo------   22.Set71! (16): New! [5 reject] (11 other internal).
+---------------------+------oo-----ooo----ooo------ooooooooo-----ooo------   23.Set5? (9): Set36(4) [2 parent; 3 reject].
+---------------------++-----oo-----ooo----ooo------ooooooooo-----ooo------   24.Set140! (16): Set96(15) [1 parent].
+---------------------+-+----oo-----ooo----ooo------ooooooooo-----ooo------   25.Set69! (26): Set36(26).
+---------------------+--+---oo-----ooo----ooo------ooooooooo-----ooo------   26.Set72! (23): Set36(23).
+----------------------ooo+--oo-----ooo----ooo------ooooooooo-----ooo------   27.Set68! (29): Set83(29).
+----------------------ooo-+-oo-----ooo----ooo------ooooooooo-----ooo------   28.Set66! (15): Set115(15).
+----------------------ooo--+-------ooo----ooo------ooooooooo-----ooo------   29.Set14? (41): Set91(13) Set133(4) [21 parent; 2 reject] (1 other internal).
+----------------------ooo--++------ooo----ooo------ooooooooo-----ooo------   30.Set119! (9): New! [2 reject] (7 other internal).
+----------------------ooo--+-+-----ooo----ooo------ooooooooo-----ooo------   31.Set65! (15): Set133(15).
+----------------------ooo---oo+----ooo----ooo------ooooooooo-----ooo------   32.Set64! (21): Set121(21).
+----------------------ooo---oo-+---ooo----ooo------ooooooooo-----ooo------   33.Set63! (20): Set65(20).
+----------------------ooo---oo--+--ooo----ooo------ooooooooo-----ooo------   34.Set62! (41): Set63(41).
+----------------------ooo---oo---+-ooo----ooo------ooooooooo-----ooo------   35.Set61! (19): Set59(19).
+----------------------ooo---oo----+-------ooo------ooooooooo-----ooo------   36.Set169? (0).
+----------------------ooo---oo----++------ooo------ooooooooo-----ooo------   37.Set167! (19): Set81(17) [2 parent].
+----------------------ooo---oo----+-+-----ooo------ooooooooo-----ooo------   38.Set166! (28): Set170(28).
+----------------------ooo---oo----+--+----ooo------ooooooooo-----ooo------   39.Set98! (22): Set124(22).
+----------------------ooo---oo-----ooo+---ooo------ooooooooo-----ooo------   40.Set56! (17): Set22(17).
+----------------------ooo---oo-----ooo-+--ooo------ooooooooo-----ooo------   41.Set161! (39): Set71(39).
+----------------------ooo---oo-----ooo--+-ooo------ooooooooo-----ooo------   42.Set54! (14): Set111(14).
+----------------------ooo---oo-----ooo---+-o-------ooooooooo-----ooo------   43.Set163? (5): (5 other internal).
+----------------------ooo---oo-----ooo---++--------ooooooooo-----ooo------   44.Set94? (40): (40 other internal).
+----------------------ooo---oo-----ooo---+++-------ooooooooo-----ooo------   45.Set102! (107): New! (107 other internal).
+----------------------ooo---oo-----ooo---+-o+------ooooooooo-----ooo------   46.Set53! (42): Set68(42).
+----------------------ooo---oo-----ooo----ooo+-----ooooooooo-----ooo------   47.Set51! (25): Set67(25).
+----------------------ooo---oo-----ooo----ooo-+----ooooooooo-----ooo------   48.Set50! (27): Set66(27).
+----------------------ooo---oo-----ooo----ooo--+---ooooooooo-----ooo------   49.Set49! (19): Set61(19).
+----------------------ooo---oo-----ooo----ooo---+--ooooooooo-----ooo------   50.Set48! (50): Set62(50).
+----------------------ooo---oo-----ooo----ooo----+-ooooooooo-----ooo------   51.Set47! (23): Set64(23).
+----------------------ooo---oo-----ooo----ooo-----+-o-oo---------ooo------   52.Set11? (154): [3 reject] (151 other internal).
+----------------------ooo---oo-----ooo----ooo-----++--oo---------ooo------   53.Set113? (30): Set51(28) [2 parent].
+----------------------ooo---oo-----ooo----ooo-----+++-oo---------ooo------   54.Set82! (0).
+----------------------ooo---oo-----ooo----ooo-----+-o+-----------ooo------   55.Set151? (0).
+----------------------ooo---oo-----ooo----ooo-----+-o++----------ooo------   56.Set137! (28): New! (28 other internal).
+----------------------ooo---oo-----ooo----ooo-----+-o+-+---------ooo------   57.Set57! (40): Set58(39) [1 parent].
+----------------------ooo---oo-----ooo----ooo-----+-o-oo+--------ooo------   58.Set45! (56): Set127(21) [35 parent].
+----------------------ooo---oo-----ooo----ooo-----+-o-oo-+-------ooo------   59.Set70! (17): Set154(17).
+----------------------ooo---oo-----ooo----ooo-----+-o-oo--+------ooo------   60.Set108! (21): Set114(21).
+----------------------ooo---oo-----ooo----ooo-----+-o-oo---+-----ooo------   61.Set77! (26): Set160(26).
+----------------------ooo---oo-----ooo----ooo------ooooooooo+----ooo------   62.Set44! (33): Set60(33).
+----------------------ooo---oo-----ooo----ooo------ooooooooo-+---ooo------   63.Set41! (79): Set31(45) Set56(32) (2 other internal).
+----------------------ooo---oo-----ooo----ooo------ooooooooo--+--ooo------   64.Set40! (24): Set119(23) (1 other internal).
+----------------------ooo---oo-----ooo----ooo------ooooooooo---+-ooo------   65.Set39! (31): Set49(31).
+----------------------ooo---oo-----ooo----ooo------ooooooooo----+---------   66.Set4? (64): Set89(16) Set98(4) [42 parent; 2 reject].
+----------------------ooo---oo-----ooo----ooo------ooooooooo----++--------   67.Set21! (13): Set89(13).
+----------------------ooo---oo-----ooo----ooo------ooooooooo----+-+-------   68.Set92! (49): Set149(19) Set98(17) [13 parent].
+----------------------ooo---oo-----ooo----ooo------ooooooooo----+--+------   69.Set38! (30): Set45(30).
+----------------------ooo---oo-----ooo----ooo------ooooooooo-----ooo+-----   70.Set36! (145): Set47(140) [1 reject] (4 other internal).
+----------------------ooo---oo-----ooo----ooo------ooooooooo-----ooo-+----   71.Set35! (457): Set48(454) [3 reject].
+----------------------ooo---oo-----ooo----ooo------ooooooooo-----ooo--+---   72.Set139! (14): Set70(14).
+----------------------ooo---oo-----ooo----ooo------ooooooooo-----ooo---+--   73.Set31! (22): Set24(22).
+----------------------ooo---oo-----ooo----ooo------ooooooooo-----ooo----+-   74.Set20! (58): Set134(26) Set171(13) [1 parent] (18 other internal).
+----------------------ooo---oo-----ooo----ooo------ooooooooo-----ooo-----+   75.Set15! (17): New! (17 other internal).
-oooooooooooooooooooooooooooooooooooooooooooooooooooooooooooooooooooooooooo   76.Reject (740): Set86(75) Set135(32) Set21(23) Set132(17) [108 parent; 301 reject] (151 other internal) + 33.
```

```
ReportA --> 51/56 (91.1%) > 50% identical (non-new) leaf sets.
ReportB --> 51/75 (68.0%) > 50% identical sets.

ReportA --> 48/56 (85.7%) > 80% identical (non-new) leaf sets.
ReportB --> 48/75 (64.0%) > 80% identical sets.

ReportC --> 46/56 (82.1%) > 90% identical (non-new) leaf sets.
ReportD --> 46/75 (61.3%) > 90% identical sets.

ReportE --> 38/56 (67.9%) identical (non-new) leaf sets.
ReportF --> 38/75 (50.7%) identical sets.
```

Figure S5. **An example output file from our simulation procedure**.

The output shown is from an analysis of C2-domains (cd00030), the hierarchy of which contained 70 nodes and 23,452 sequences. We applied the amcBPPS program to simulated sequences generated using the profile HMMs associated with the nodes of the C2 hierarchy; for each node we generated the same number of aligned sequences as were assigned to that node. The amcBPPS program returned the hierarchy shown directly below. (The source hierarchy is shown on the subsequent page.) This reveals that 52 out of the 57 sequence sets in the new hierarchy (or 91% of them) precisely correspond to the profiles from which those sequences were generated. (On each line of the FD-table the number of sequences generated from each of the HMM profiles is indicated in parentheses following the corresponding source node identifier).

FD-table (found):

```
+---------oo-------------------------oooooo-------------- 1.Set71(12116) =  Set121(4177) Set44(52) Set108(13) Set104(120) Set46(3187) Set117(168) Set14(1) Set99(3406) + 16 other sequences.
++--------oo-------------------------oooooo-------------- 2.Set18(1) Set91(239) Set80(21) Set96(5) Set4(377) Set8(176) Random(157)
+-+-------oo-------------------------oooooo-------------- 3.Set58(114) =  Set68(10) Set112(104)
+--+------oo-------------------------oooooo-------------- 4.Set44(177) =  Set75(177)
+---+-----oo-------------------------oooooo-------------- 5.Set42(103) =  Set17(103)
+----+----oo-------------------------oooooo-------------- 6.Set28(72) =  Set92(72)
+-----+---oo-------------------------oooooo-------------- 7.Set7(172) =  Set18(34) Set78(138)
+------+--oo-------------------------oooooo-------------- 8.Set59(50) =  Set97(50)
+-------+-oo-------------------------oooooo-------------- 9.Set57(63) =  Set35(63)
+--------+---------------------------oooooo-------------- 10.Set55(86) =  Set94(86)
+--------++--------------------------oooooo-------------- 11.Set1(17) =  Set2(17)
+--------+-+-------------------------oooooo-------------- 12.Set52(96) =  Set63(96)
+---------oo+------------------------oooooo-------------- 13.Set50(65) =  Set62(65)
+---------oo-+-----------------------oooooo-------------- 14.Set49(79) =  Set65(79)
+---------oo--+----------------------oooooo-------------- 15.Set48(132) =  Set102(132)
+---------oo---+---------------------oooooo-------------- 16.Set47(48) =  Set37(48)
+---------oo----+--------------------oooooo-------------- 17.Set46(104) =  Set85(104)
+---------oo-----+-------------------oooooo-------------- 18.Set45(69) =  Set93(69)
+---------oo------+------------------oooooo-------------- 19.Set41(75) =  Set33(75)
+---------oo-------+-----------------oooooo-------------- 20.Set40(73) =  Set83(73)
+---------oo--------+----------------oooooo-------------- 21.Set39(40) =  Set59(40)
+---------oo---------+---------------oooooo-------------- 22.Set38(37) =  Set87(37)
+---------oo----------+--------------oooooo-------------- 23.Set37(98) =  Set40(98)
+---------oo-----------+-------------oooooo-------------- 24.Set36(73) =  Set66(73)
+---------oo------------+------------oooooo-------------- 25.Set35(60) =  Set106(60)
+---------oo-------------+-----------oooooo-------------- 26.Set34(108) =  Set118(108)
+---------oo--------------+----------oooooo-------------- 27.Set33(64) =  Set113(64)
+---------oo---------------+---------oooooo-------------- 28.Set32(105) =  Set27(105)
+---------oo----------------+--------oooooo-------------- 29.Set31(58) =  Set82(58)
+---------oo-----------------+-------oooooo-------------- 30.Set30(45) =  Set23(45)
+---------oo------------------+------oooooo-------------- 31.Set27(577) =  Set72(577)
+---------oo-------------------+-----oooooo-------------- 32.Set26(481) =  Set21(481)
+---------oo--------------------+----oooooo-------------- 33.Set25(62) =  Set100(62)
+---------oo---------------------+---oooooo-------------- 34.Set24(137) =  Set31(137)
+---------oo----------------------+--oooooo-------------- 35.Set22(272) =  Set109(272)
+---------oo-----------------------+-oooooo-------------- 36.Set21(201) =  Set25(201)
+---------oo------------------------+---ooo-------------- 37.Set67(520) =  Set121(1) Set18(1) Set80(74) Set78(3) Set4(396) Set53(45)
+---------oo------------------------++--ooo-------------- 38.Set54(156) =  Set69(156)
+---------oo------------------------+-+-ooo-------------- 39.Set20(127) =  Set73(127)
+---------oo------------------------+--+----------------- 40.Set63(63) =  Set80(63)
+---------oo------------------------+--++---------------- 41.Set53(228) =  Set13(228)
+---------oo------------------------+--+-+--------------- 42.Set51(131) =  Set67(131)
+---------oo------------------------+--+--+-------------- 43.Set43(268) =  Set18(268)
+---------oo-------------------------oooooo+------------- 44.Set19(193) =  Set38(193)
+---------oo-------------------------oooooo-+------------ 45.Set18(195) =  Set15(195)
+---------oo-------------------------oooooo--+----------- 46.Set17(321) =  Set24(321)
+---------oo-------------------------oooooo---+---------- 47.Set16(106) =  Set26(106)
+---------oo-------------------------oooooo----+--------- 48.Set15(35) =  Set103(35)
+---------oo-------------------------oooooo-----+-------- 49.Set14(116) =  Set29(116)
+---------oo-------------------------oooooo------+------- 50.Set13(364) =  Set14(364)
+---------oo-------------------------oooooo-------+------ 51.Set12(1549) =  Set96(1549)
+---------oo-------------------------oooooo--------+----- 52.Set11(148) =  Set34(148)
+---------oo-------------------------oooooo---------+---- 53.Set10(282) =  Set71(282)
+---------oo-------------------------oooooo----------+--- 54.Set9(407) =  Set116(407)
+---------oo-------------------------oooooo-----------+-- 55.Set8(130) =  Set19(130)
+---------oo-------------------------oooooo------------+- 56.Set4(158) =  Set61(158)
+---------oo-------------------------oooooo-------------+ 57.Set2(321) =  Set28(321)
-oooooooooooooooooooooooooooooooooooooooooooooooooooooooo 58.Random(1505) =  Set121(220) Set44(84) Set68(16) Set108(56) Set46(60) Set14(13) Set99(40) Set28(2) Set21(3)  Set4(3) Random(1008)
```

Figure S5 (cont).

FD-table (source):

```
+-----------ooo-----oooooooooooooo----------oo-----------------ooooo-- 1.Set121?
++----------ooo-----oooooooooooooo----------oo-----------------ooooo-- 2.Set61!
+-+---------ooo-----oooooooooooooo----------oo-----------------ooooo-- 3.Set100!
+--+--------ooo-----oooooooooooooo----------oo-----------------ooooo-- 4.Set92!
+---+-------ooo-----oooooooooooooo----------oo-----------------ooooo-- 5.Set72!
+----+------ooo-----oooooooooooooo----------oo-----------------ooooo-- 6.Set65!
+-----+-----ooo-----oooooooooooooo----------oo-----------------ooooo-- 7.Set38!
+------+----ooo-----oooooooooooooo----------oo-----------------ooooo-- 8.Set24!
+-------+---ooo-----oooooooooooooo----------oo-----------------ooooo-- 9.Set118!
+--------+--ooo-----oooooooooooooo----------oo-----------------ooooo-- 10.Set116!
+---------+-ooo-----oooooooooooooo----------oo-----------------ooooo-- 11.Set109!
+----------+-oo-----oooooooooooooo----------oo-----------------ooooo-- 12.Set44?
+----------++-------oooooooooooooo----------oo-----------------ooooo-- 13.Set68?
+----------+++------oooooooooooooo----------oo-----------------ooooo-- 14.Set112!
+----------++-+-----oooooooooooooo----------oo-----------------ooooo-- 15.Set108!
+-----------ooo+----oooooooooooooo----------oo-----------------ooooo-- 16.Set106!
+-----------ooo-+---oooooooooooooo----------oo-----------------ooooo-- 17.Set104!
+-----------ooo--+--oooooooooooooo----------oo-----------------ooooo-- 18.Set103!
+-----------ooo---+-oooooooooooooo----------oo-----------------ooooo-- 19.Set97!
+-----------ooo----+----oooo----------------oo-----------------ooooo-- 20.Set46?
+-----------ooo----++---oooo----------------oo-----------------ooooo-- 21.Set117!
+-----------ooo----+-+--oooo----------------oo-----------------ooooo-- 22.Set94!
+-----------ooo----+--+-oooo----------------oo-----------------ooooo-- 23.Set14!
+-----------ooo----+---+--------------------oo-----------------ooooo-- 24.Set99?
+-----------ooo----+---++-------------------oo-----------------ooooo-- 25.Set95!
+-----------ooo----+---+-+------------------oo-----------------ooooo-- 26.Set75!
+-----------ooo----+---+--+-----------------oo-----------------ooooo-- 27.Set76!
+-----------ooo----+---+---+----------------oo-----------------ooooo-- 28.Set18!
+-----------ooo----+----oooo+---------------oo-----------------ooooo-- 29.Set35!
+-----------ooo----+----oooo-+--------------oo-----------------ooooo-- 30.Set66!
+-----------ooo----+----oooo--+-------------oo-----------------ooooo-- 31.Set77!
+-----------ooo----+----oooo---+------------oo-----------------ooooo-- 32.Set67!
+-----------ooo----+----oooo----+-----------oo-----------------ooooo-- 33.Set102!
+-----------ooo----+----oooo-----+----------oo-----------------ooooo-- 34.Set113!
+-----------ooo-----oooooooooooooo+---------oo-----------------ooooo-- 35.Set93!
+-----------ooo-----oooooooooooooo-+--------oo-----------------ooooo-- 36.Set91!
+-----------ooo-----oooooooooooooo--+-------oo-----------------ooooo-- 37.Set85!
+-----------ooo-----oooooooooooooo---+------oo-----------------ooooo-- 38.Set83!
+-----------ooo-----oooooooooooooo----+-----oo-----------------ooooo-- 39.Set82!
+-----------ooo-----oooooooooooooo-----+----oo-----------------ooooo-- 40.Set80!
+-----------ooo-----oooooooooooooo------+---oo-----------------ooooo-- 41.Set78!
+-----------ooo-----oooooooooooooo-------+--oo-----------------ooooo-- 42.Set73!
+-----------ooo-----oooooooooooooo--------+-oo-----------------ooooo-- 43.Set71!
+-----------ooo-----oooooooooooooo---------+-------------------ooooo-- 44.Set2?
+-----------ooo-----oooooooooooooo---------++------------------ooooo-- 45.Set63!
+-----------ooo-----oooooooooooooo---------+-+-----------------ooooo-- 46.Set62!
+-----------ooo-----oooooooooooooo----------oo+----------------ooooo-- 47.Set59!
+-----------ooo-----oooooooooooooo----------oo-+---------------ooooo-- 48.Set40!
+-----------ooo-----oooooooooooooo----------oo--+--------------ooooo-- 49.Set37!
+-----------ooo-----oooooooooooooo----------oo---+-------------ooooo-- 50.Set34!
+-----------ooo-----oooooooooooooo----------oo----+------------ooooo-- 51.Set33!
+-----------ooo-----oooooooooooooo----------oo-----+-----------ooooo-- 52.Set31!
+-----------ooo-----oooooooooooooo----------oo------+----------ooooo-- 53.Set29!
+-----------ooo-----oooooooooooooo----------oo-------+---------ooooo-- 54.Set28!
+-----------ooo-----oooooooooooooo----------oo--------+--------ooooo-- 55.Set27!
+-----------ooo-----oooooooooooooo----------oo---------+-------ooooo-- 56.Set26!
+-----------ooo-----oooooooooooooo----------oo----------+------ooooo-- 57.Set25!
+-----------ooo-----oooooooooooooo----------oo-----------+-----ooooo-- 58.Set23!
+-----------ooo-----oooooooooooooo----------oo------------+----ooooo-- 59.Set21!
+-----------ooo-----oooooooooooooo----------oo-------------+---ooooo-- 60.Set19!
+-----------ooo-----oooooooooooooo----------oo--------------+--ooooo-- 61.Set96!
+-----------ooo-----oooooooooooooo----------oo---------------+-ooooo-- 62.Set15!
+-----------ooo-----oooooooooooooo----------oo----------------+------- 63.Set4?
+-----------ooo-----oooooooooooooo----------oo----------------++------ 64.Set69!
+-----------ooo-----oooooooooooooo----------oo----------------+-+----- 65.Set17!
+-----------ooo-----oooooooooooooo----------oo----------------+--+---- 66.Set13!
+-----------ooo-----oooooooooooooo----------oo----------------+---+--- 67.Set107!
+-----------ooo-----oooooooooooooo----------oo----------------+----+-- 68.Set53!
+-----------ooo-----oooooooooooooo----------oo-----------------ooooo+- 69.Set8!
+-----------ooo-----oooooooooooooo----------oo-----------------ooooo-+ 70.Set87!
-ooooooooooooooooooooooooooooooooooooooooooooooooooooooooooooooooooooo 71.Random=7818.
```

Figure S6. **A CDD "sequence tree" computed from the amcBPPS hierarchy for RRM domains**.
This screen shot shows part of a "sequence tree" computed from the input sequences used for the amcBPPS analysis of RRM domains shown in Table 3. The pair-wise sequence similarities used to construct the tree were calculated using a version of the gapped BLAST program termed pBLAST. The tree is colored according to the sub-groups in the automatically generated hierarchy.

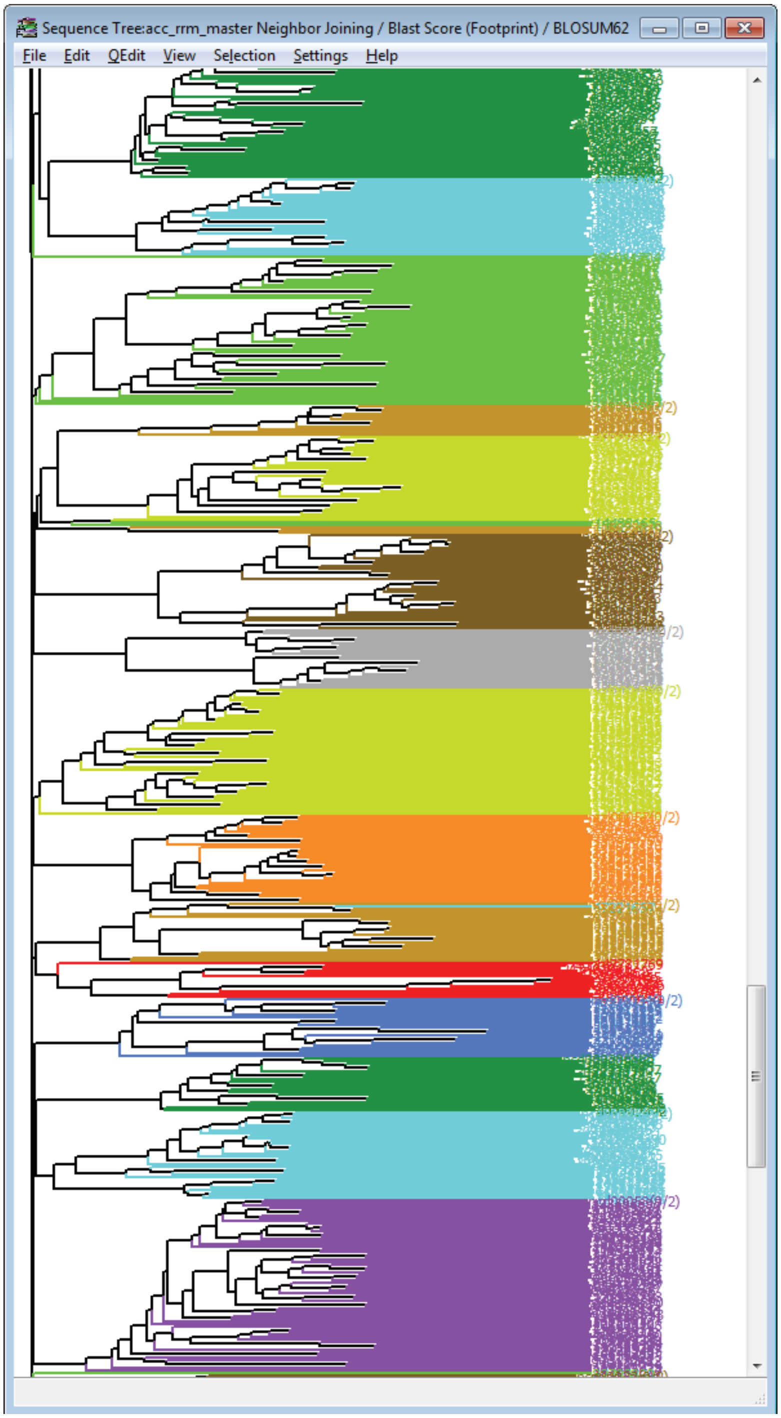

Supplement: Additional File 1 — Additional figures referred to in the main article as Figures S1–S6. [file 1471-2105-13-144-S1.pdf]
